# Self-assembling SARS-CoV-2 spike-HBsAg nanoparticles elicit potent and durable neutralizing antibody responses via genetic delivery

**DOI:** 10.1038/s41541-023-00707-w

**Published:** 2023-08-08

**Authors:** Cuiping Liu, Lingshu Wang, Jonah S. Merriam, Wei Shi, Eun Sung Yang, Yi Zhang, Man Chen, Wing-Pui Kong, Cheng Cheng, Yaroslav Tsybovsky, Tyler Stephens, Raffaello Verardi, Kwanyee Leung, Cody Stein, Adam S. Olia, Darcy R. Harris, Misook Choe, Baoshan Zhang, Barney S. Graham, Peter D. Kwong, Richard A. Koup, Amarendra Pegu, John R. Mascola

**Affiliations:** 1 Vaccine Research Center, National Institute of Allergy and Infectious Diseases, NIH, Bethesda, MD 20892 USA; 2 Vaccine Research Center Electron Microscopy Unit, Cancer Research Technology Program, Leidos Biomedical Research, Inc., Frederick National Laboratory for Cancer Research, Frederick, MD 21702 USA

**Keywords:** Translational research, Infectious diseases

## Abstract

While several COVID-19 vaccines have been in use, more effective and durable vaccines are needed to combat the ongoing COVID-19 pandemic. Here, we report highly immunogenic self-assembling SARS-CoV-2 spike-HBsAg nanoparticles displaying a six-proline-stabilized WA1 (wild type, WT) spike S6P on a HBsAg core. These S6P-HBsAgs bound diverse domain-specific SARS-CoV-2 monoclonal antibodies. In mice with and without a HBV pre-vaccination, DNA immunization with S6P-HBsAgs elicited significantly more potent and durable neutralizing antibody (nAb) responses against diverse SARS-CoV-2 strains than that of soluble S2P or S6P, or full-length S2P with its coding sequence matching mRNA-1273. The nAb responses elicited by S6P-HBsAgs persisted substantially longer than by soluble S2P or S6P and appeared to be enhanced by HBsAg pre-exposure. These data show that genetic delivery of SARS-CoV-2 S6P-HBsAg nanoparticles can elicit greater and more durable nAb responses than non-nanoparticle forms of stabilized spike. Our findings highlight the potential of S6P-HBsAgs as next generation genetic vaccine candidates against SARS-CoV-2.

## Introduction

Since the first known case of Coronavirus disease 2019 (COVID-19) identified in December of 2019, COVID-19 infection has been detected in over 500 million people and caused 6 million deaths worldwide (https://coronavirus.jhu.edu/map.html). COVID-19 is caused by severe acute respiratory syndrome coronavirus 2 (SARS-CoV-2), a beta-coronavirus closely related to SARS^1^. The SARS-CoV-2 spike is a trimeric glycoprotein that coats the surface of SARS-CoV-2 viral particles^2^ and consists of an N-terminal signal peptide, S1 and S2 subunits^3,4^. The S1 subunit contains an N-terminal domain (NTD) and a receptor-binding domain (RBD). The RBD domain is responsible for the binding to human angiotensin-converting enzyme 2 (ACE2), the major receptor in humans for SARS-CoV-2. The S1 subunit also contains SD1 and SD2 domains, which are located between the RBD and the furin cleavage site. The S2 subunit is composed of the fusion peptide (FP), heptapeptide repeat sequences 1 and 2 (HR1 and HR2), transmembrane domain (TM), and cytoplasmic domain^4^. The S2 subunit is involved in membrane fusion. Owing to its role in receptor binding, viral attachment, and entry into host cells, SARS-CoV-2 spike is the target of many authorized or licensed COVID-19 vaccines^5^.

SARS-CoV-2 spike exists in a metastable prefusion conformation that spontaneously transitions to its post-fusion conformation^3^. Two-proline substitutions, K986P and V987P, located between the HR1 and HR2 sequences, can stabilize the spike in its prefusion conformation^2^. The SARS-CoV-2 spike stabilized by these two-proline substitutions, named SARS-CoV-2 S2P, is the protein encoded by the mRNA vaccines from Moderna and Pfizer-BioNTech^6,7^. Additional mutations including F817P, A892P, A899P and A942P can further stabilize the prefusion conformation of SARS-CoV-2 S2P^8^. The resulting spike was named SARS-CoV-2 S6P and has been shown to be more immunogenic and protective than SARS-CoV-2 S2P^9^. In some alternative vaccine designs, SARS-CoV-2 S6P has elicited potent nAb responses against SARS-CoV-1^10^.

Among the leading COVID-19 vaccines, the mRNA vaccines from both Moderna and Pfizer-BioNTech exhibit high protective efficacy^11^. However, the protection offered by these vaccines has been waning over time^12,13^ and has declined considerably against the Delta and Omicron sublineages, as compared to the ancestral Wuhan or WA1 strain^14,15^. A third dose of the mRNA vaccines is needed to achieve high level of protection against Omicron variants and a fourth dose of the mRNA vaccines is sometimes recommended to restore the antibody levels and improve clinical protection^16^. Recently, the bivalent mRNA vaccines encoding both WA1 and BA.4/5 S2Ps from Moderna and Pfizer-BioNTech have been authorized as a booster shot (https://www.fda.gov/news-events/press-announcements/coronavirus-covid-19-update-fda-authorizes-moderna-pfizer-biontech-bivalent-covid-19-vaccines-use). While mRNA vaccines have been proven to be effective, there remains room to improve the potency and durability of the elicited immune response.

Nanoparticle vaccine platforms allow multivalent antigen presentation and can elicit more potent immune responses than protein immunogens^17^. Several nanoparticle platforms using lumazine synthase, ferritin or I53-50 displaying SARS-CoV-2 spike or its RBD domain elicit potent nAb responses against SARS-CoV-2^18–21^. HBsAg can self-assemble into a 22 nm nanoparticle and has been used successfully in multiple vaccines including Hepaccine B and Recombivax HB^22^. Recently, a HBsAg-based malaria vaccine, R21/MM, has shown 77% efficacy in a phase IIb trial and advanced into phase III^23^. HBsAg has also been used in recent vaccine designs displaying SARS-CoV-2 RBD^24–26^. In addition, the hepatitis B vaccine can elicit over 30 years of immunological memory against HBsAg in healthy people after the first vaccination at the age of over 6 months, without the need of a booster shot^27,28^. In this study, we designed and characterized self-assembling HBsAg-based nanoparticles displaying the SARS-CoV-2 spike and compared the immunogenicity of these spike-HBsAg nanoparticles and non-nanoparticle form of stabilized spikes in mice via genetic delivery.

## Results

### SARS-CoV-2 S6P-HBsAg constructs can express self-assembling nanoparticles

**Fig. 1:**
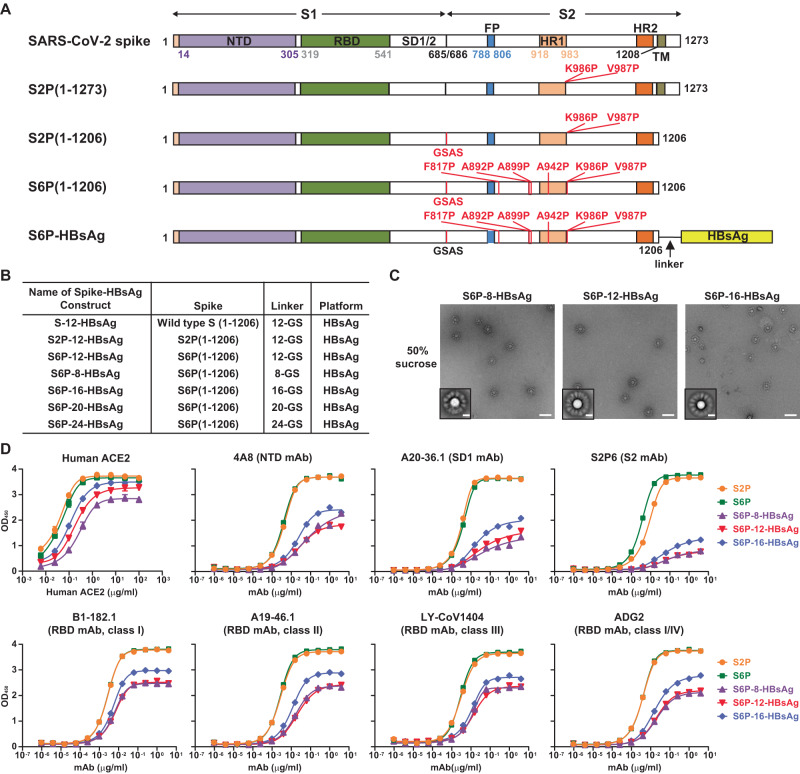
Self-assembling SARS-CoV-2 S6P-HBsAg nanoparticles. **A** Schematic representation of full-length SARS-CoV-2 WA1 spike, S2P(1-1273), S2P(1-1206), S6P(1-1206) and S6P-HBsAg fusion protein. The individual domain of the spike or its ectodomains was colored and indicated. S2P(1-1273) is full-length spike harboring K986P and V987P mutations. S2P(1-1206) and S6P(1-1206) represent the ectodomains of S2P and S6P spanning from amino acids 1 to 1206, respectively. S6P(1-1206) contains six-proline substitutions: F817P, A892P, A899P, A942P, K986P and V987P. Both S2P(1-1206) and S6P(1-1206) contain RRAR-to-GSAS substitution at the furin cleavage site. S6P-HBsAg fusion was constructed by joining S6P(1-1206) and HBsAg from amino acid 1 to 226 with a linker of GS repeats. **B** Constructs tested for nanoparticle formation on a HBsAg core. The spike-HBsAg constructs for SARS-CoV-2 WA1 spike, S2P and S6P with a 12-GS linker were named S-12-HBsAg, S2P-12-HBsAg and S6P-12-HBsAg, respectively. Varying linkers of 8- to 24-GS were tested and named accordingly. **C** Negative-stain EM images of S6P-8-HBsAg (left), S6P-12-HBsAg (middle) and S6P-16-HBsAg (right) from the 50% sucrose fractions. The insets show 2D class averages of corresponding nanoparticle constructs. Scale bars represent 100 nm in micrographs and 20 nm in 2D class average images. **D** Human ACE2 binding and antigenicity of S6P-HBsAgs. Plates were coated with 1 µg/ml of SARS-CoV-2 S2P, S6P or S6P-HBsAg nanoparticles. Human ACE2 was assessed at a concentration ranging from 6.1 ng/ml to 100 µg/ml. The mAbs specific to the NTD, SD1, S2 or RBD domain of SARS-CoV-2 spike were tested from 1 pg/ml to 4 µg/ml. All plots were color-coded according to the legends on the right.

We designed plasmid DNAs encoding fusion proteins of SARS-CoV-2 WA1 spike, S2P and S6P, which consist of the corresponding ectodomain (amino acids (aa) 1-1206) fused to HBsAg (aa 1-226) by GS linkers of varying lengths (Fig. 1A, B). After transfection into Expi293 cells, these constructs expressed proteins that can be detected in the 30% to 65% sucrose fractions after ultracentrifugation using a sucrose gradient. These fractions from all tested constructs showed binding to anti-HBsAg antibody and four mAbs specific to SARS-CoV-2 spike: NTD mAb S652-118, RBD mAbs A23-58.1 and LY-CoV555, and S2 mAb WS6^29–32^ (Supplementary Fig. 1, shown only for S6P-HBsAgs). A relatively smaller proportion of well-formed nanoparticles were observed in the sucrose fractions obtained after transfection with DNA encoding WA1 spike-12-HBsAg and S2P-12-HBsAg. In contrast, S6P-8-HBsAg, S6P-12-HBsAg, and S6P-16-HBsAg from the 50% sucrose fractions formed well-defined nanoparticles (Fig. 1C). These nanoparticles had diameters of ~70 nm, with a yield of 12 to 16 mg/L for each construct. The 2D class averages of purified S6P-12-HBsAg and S6P-16-HBsAg showed mushroom-shaped S6P, suggesting that, in these HBsAg nanoparticles, the S6P ectodomain in its prefusion conformation^2,33^ was displayed on the surface.

### SARS-CoV-2 S6P-HBsAg nanoparticles can bind human ACE2 and diverse SARS-CoV-2 mAbs

To characterize these SARS-CoV-2 S6P-HBsAg nanoparticles, we tested the binding of purified S6P-8-HBsAg, S6P-12-HBsAg and S6P-16-HBsAg nanoparticles to human ACE2, as ACE2 is one of the primary receptors for SARS-CoV-2^34^. WA1 S2P and S6P recombinant proteins were used as controls, as they adopt stabilized prefusion conformation^2,8^. While S2P and S6P bound to human ACE2 with similar patterns (Fig. 1D), all three S6P-HBsAgs showed moderately weaker binding to human ACE2 than S2P and S6P. Among these three types of nanoparticles, S6P-8-HBsAg appeared to be the weakest binder and S6P-16-HBsAg was the strongest binder.

To monitor whether individual domains of SARS-CoV-2 spike within the nanoparticles are accessible, we evaluated the antigenicity of these three S6P-HBsAgs by both ELISA and Bio-Layer Interferometry (BLI). mAbs specific to the NTD, SD1, S2 and RBD domains of SARS-CoV-2 spike were used for this evaluation (Supplementary Table 1). In ELISA assays, S2P and S6P showed nearly identical binding profiles to NTD mAbs 4A8 and 4-8, SD1 mAb A20-36.1, and all tested RBD mAbs (Fig. 1D and Supplementary Fig. 2). S6P exhibited marginally higher affinities to NTD mAbs S652-118, 4-19 and 1-68, S2 mAbs S2P6 and WS6, and remarkably higher affinity to S2 mAb S652-112 than S2P. Compared with S2P and S6P, the three S6P-HBsAgs showed weaker binding by ELISA to all tested mAbs specific to the NTD, SD1, S2 and RBD domains of SARS-CoV-2 spike. While S6P-8-HBsAg and S6P-12-HBsAg showed similar binding affinities to most tested mAbs, S6P-8-HBsAg exhibited slightly lower affinities to NTD mAbs S652-118, 4-19 and 1-68, SD1 mAb A20-36.1, and S2 mAb S652-112. (The references for these mAbs are listed in Supplementary Table 1). Among these three types of nanoparticles, S6P-16-HBsAg exhibited the highest affinities to all tested mAbs specific to the NTD, SD1, S2 and RBD domains of SARS-CoV-2 spike. An additional control experiment showed that these SARS-CoV-2 mAbs do not exhibit non-specific binding to HBsAg (Supplementary Fig. 3). In BLI assays, compared with the affinity of free S6P, the S6P displayed on a HBsAg core showed comparable or stronger affinity to the majority of the tested mAbs (Supplementary Fig. 4 and Supplementary Table 2). Of note, while SARS-CoV-2 S2, S2P and S6P showed binding to S2 mAbs CoVA1-07, CoVA2-14 and CoVA2-18^35^, the S6P displayed on the HBsAg nanoparticle did not bind these three S2 mAbs under the tested condition. S6P-12-HBsAg and S6P-16-HBsAg exhibited stronger affinity than S6P-8-HBsAg for most of the tested RBD mAbs. Based on these antigenicity data, we chose S6P-12-HBsAg and S6P-16-HBsAg for immunogenicity studies.

### DNA encoding SARS-CoV-2 S6P-HBsAgs elicits potent binding and neutralizing antibody responses against WA1

**Fig. 2:**
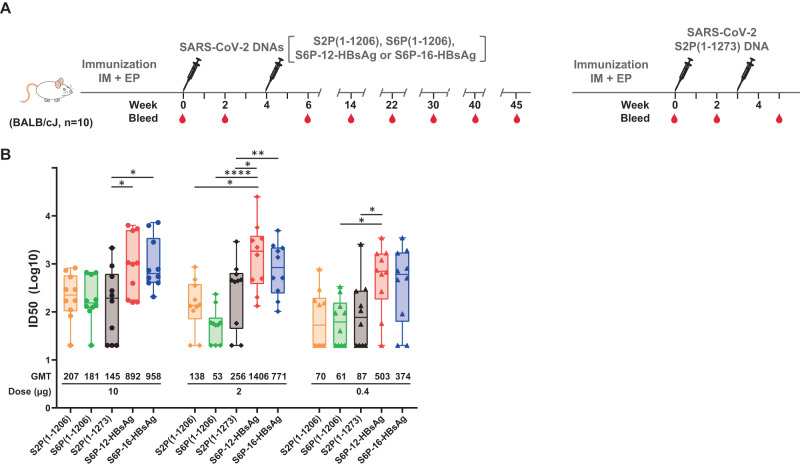
Neutralization potency elicited by SARS-CoV-2 S6P-HBsAgs. **A** Immunization schema of SARS-CoV-2 DNA constructs. SARS-CoV-2 WA1 S2P(1-1206), S6P(1-1206), S6P-12-HBsAg, S6P-16-HBsAg and S2P(1-1273) DNA matching mRNA-1273 sequence were evaluated. A total of 10, 2, or 0.4 µg of each DNA was injected intramuscularly to two hind legs of BALB/cJ mice at week 0 and 4, excepting S2P(1-1273), which was administer at week 0 and 3. Electroporation was applied following each immunization. Sera were collected 2 weeks post each immunization and followed by a 5- to 10-week interval for mice immunized with S2P(1-1206), S6P(1-1206), S6P-12-HBsAg or S6P-16-HBsAg. **B** Neutralization ID50s against WA1 pseudovirus. The sera at 2 weeks post the second immunization were assessed. The ID50s were plotted in the box and whiskers format, in which, the median ID50 was indicated by a horizontal line, and the data points in the median quartile of each group were boxed. The error bars represent 95% confidence interval. The immunogens and doses were indicated. The neutralization ID50s were plotted in a logarithmic scale as dots, diamonds and triangles for 10, 2, and 0.4 µg doses, respectively. The data points were colored in beige, green, black, red and blue for S2P(1-1206), S6P(1-1206), S2P(1-1273), S6P-12-HBsAg and S6P-16-HBsAg, respectively. The geometric mean titers were listed below each group. The statistical analyses were performed using the two-way ANOVA test for S2P(1-1206), S6P(1-1206), S6P-12-HBsAg and S6P-16-HBsAg following log transformation of the data. The comparison between the same dose of S6P-HBsAg and S2P(1-1273) was done using the two tailed Mann-Whitney test with Dunn’s multiple comparisons test. *p < 0.05; **p < 0.01; ***p < 0.001; ****p < 0.0001. Full statistical analyses were shown in Supplementary Table 4.

To assess the immunogenicity of S6P-12-HBsAg and S6P-16-HBsAg, we immunized 6- to 8-week-old BALB/cJ mice with plasmid DNA encoding S6P-12-HBsAg or S6P-16-HBsAg, using plasmid DNA encoding S2P(1-1206), S6P(1-1206) and S2P(1-1273), respectively, as controls (Fig. 2A). Each animal received electroporation following DNA immunization. S2P(1-1273) is the full-length S2P with its coding sequence matching mRNA-1273. As our HBsAg construct did not express well-defined nanoparticles, we did not use HBsAg as a negative control in the animal study. With two intramuscular immunizations plus electroporation spaced 4-week apart, 10, 2, and 0.4 µg of S6P-12-HBsAg or S6P-16-HBsAg elicited potent binding antibodies to WA1 S2P, RBD and NTD domains (Supplementary Fig. 5). The geometric mean titers (GMTs) increased from week 2 to week 6. The GMTs against S2P or RBD elicited by S6P-HBsAgs were comparable to those elicited by the same dose of non-nanoparticle forms of stabilized spikes S2P(1-1206), S6P(1-1206) and S2P(1-1273) at week 6. 0.4 µg of S6P-HBsAg elicited lower GMTs than the same dose of soluble S2P and S6P. Ten, 2, or 0.4 µg of S6P-12-HBsAg or S6P-16-HBsAg elicited significant higher ratios of anti-RBD titer to anti-S2P titer than the same dose of stabilized spikes. Due to the limited volume of sera available from mice immunized with S2P(1-1273), we did not perform ELISA binding to RBD or NTD for these sera.

The neutralization potency elicited by SARS-CoV-2 S6P-HBsAg nanoparticles was tested using lentiviral-based WA1 pseudovirus in sera collected at 2 weeks post the second immunization. The same dose of S2P(1-1206), S6P(1-1206) and S2P(1-1273) matching the sequence of mRNA-1273 elicited comparable ID50s (Fig. 2B). Ten, 2, and 0.4 µg S6P-12-HBsAg or S6P-16-HBsAg DNA elicited 3- to 26.5-fold higher ID50s than the same dose of these three non-nanoparticle forms of stabilized spikes (Supplementary Table 3). Two and 0.4 µg S6P-12-HBsAg or S6P-16-HBsAg DNA elicited significantly higher geometric mean ID50s than those elicited by the same or higher dose of non-nanoparticle forms of stabilized spikes (Fig. 2B and Supplementary Table 4). Similar results were obtained for ID80s against WA1 pseudovirus (Supplementary Fig. 6).

### DNA encoding SARS-CoV-2 S6P-HBsAgs elicits potent binding and neutralizing antibody responses against SARS-CoV-2 variants

As SARS-CoV-2 variants D614G, B.1.351 (Beta), B.1.617.2 (Delta) and B.1.1.529 (Omicron BA.1) pose greater risk to public health than WA1^36^, we also tested whether S6P-HBsAgs can elicit antibody responses against these variants. S6P-12-HBsAg and S6P-16-HBsAg elicited binding antibody responses to B.1.351, B.1.617.2 and B.1.1.529 S2Ps in a dose-dependent manner at both weeks 2 and 6 (Supplementary Fig. 7). The GMTs increased from week 2 to week 6. At each time point, 0.4 µg S6P-HBsAgs elicited substantially lower ELISA binding GMTs against all three variant S2Ps than the same dose of soluble S2P or S6P.

**Fig. 3:**
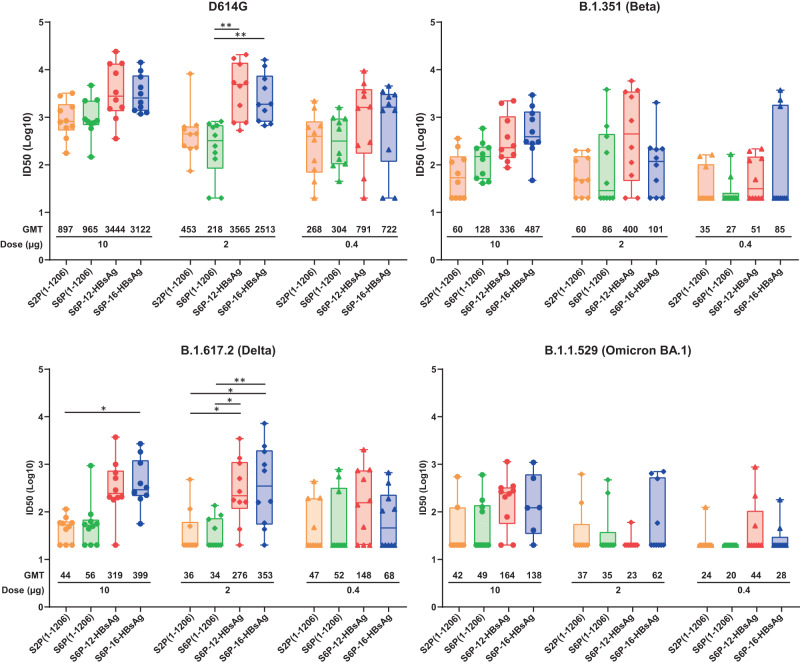
Neutralization potency against SARS-CoV-2 variant pseudovriuses elicited by SARS-CoV-2 S6P-HBsAgs. The sera at 2 weeks post the second immunization with SARS-CoV-2 S2P(1-1206), S6P(1-1206), S6P-12-HBsAg or S6P-16-HBsAg were evaluated against SARS-CoV-2 D614G, B.1.351, B.1.617.2 and B.1.1.529 pseudoviruses. The ID50s were plotted in the box and whiskers format. The median ID50 was indicated by a horizontal line, and the data points in the median quartile of each group were boxed. The error bars represent 95% confidence interval. The immunogens and doses were indicated. The ID50s were plotted in a logarithmic scale as dot, diamond or triangle for 10, 2, and 0.4 µg doses, respectively. The data points were shown in beige, green, red and blue for S2P(1-1206), S6P(1-1206), S6P-12-HBsAg and S6P-12-HBsAg, respectively. The geometric mean ID50 for each group was listed below each group. The statistical analyses were performed using the two-way ANOVA test following log transformation of the data. *p < 0.05; **p < 0.01.

Ten, 2, and 0.4 µg of soluble S2P and S6P elicited similar levels of neutralization potency against each individual pseudovirus at each dose (Fig. 3 and Supplementary Table 5). At all three doses, S6P-12-HBsAg or S6P-16-HBsAg elicited substantially higher geometric mean ID50s against D614G, B.1.351 and B.1.617.2 pseudoviruses than the same dose of soluble S2P and S6P. Two µg of S6P-12-HBsAg and 10 or 2 µg S6P-16-HBsAg elicited significantly higher neutralization potency than the same or higher doses of soluble S2P or S6P for D614G and B.1.617.2 pseudoviruses (Fig. 3 and Supplementary Table 6). For B.1.1.529 pseudovirus, S6P-HBsAgs elicited marginally increased geometric mean ID50s compared with soluble S2P and S6P. Similar results were obtained for ID80 titers (Supplementary Fig. 6).

### DNA encoding SARS-CoV-2 S6P-HBsAgs elicits durable nAb responses

As S6P-HBsAgs elicited potent nAb responses at week 6 against SARS-CoV-2 WA1 and diverse variant pseudoviruses, we assessed the durability of the neutralization potency elicited by S6P-HBsAg. At week 14, the neutralization ID50s and ID80s elicited by S6P-12-HBsAg or S6P-16-HBsAg were maintained at significantly higher levels than those elicited by the same or higher dose of soluble S2P and S6P (Supplementary Fig. 8). The ID50s and ID80s elicited by these S6P-HBsAgs at week 14 are comparable to those at week 6. Notably, the ID50s elicited by 0.4 µg of S6P-12-HBsAg or S6P-16-HBsAg increased from week 6 to week 14, though without statistical significance.

We further monitored binding antibodies in the animal sera to WA1 S2P and HBsAg from week 0 to week 45. Potent anti-WA1 S2P antibodies were detected from week 6 to week 45, for mice immunized twice with S2P(1-1206), S6P(1-1206), S6P-12-HBsAg and S6P-16-HBsAg at 10, 2, and 0.4 µg doses (Supplementary Fig. 9). Neither soluble spike groups nor nanoparticle groups showed significant change in the levels of S2P-binding antibodies over this duration. The anti-HBsAg binding antibodies were only detected from week 6 to week 45 in 10 and 2 µg of S6P-HBsAg groups, with significantly lower titers than the anti-S2P antibodies (Supplementary Figs. 9 and 10).

**Fig. 4:**
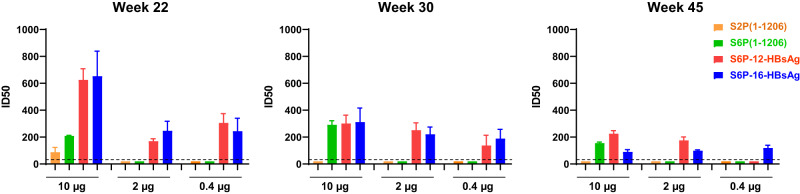
Durability of neutralizing antibody responses elicited by SARS-CoV-2 S6P-HBsAgs. The sera from mice immunized with DNA encoding SARS-CoV-2 S2P(1-1206), S6P(1-1206), S6P-12-HBsAg or S6P-16-HBsAg were tested from week 22 to week 45. The ID50s against WA1 pseudovirus obtained from the pooled serum of each group were shown for the 10, 2 and 0.4 µg doses. The bars were colored in beige, green, red and blue for S2P(1-1206), S6P(1-1206), S6P-12-HBsAg and S6P-16-HBsAg, respectively. The graphs for weeks 22, 30 and 45 were shown in different panels. The dashed lines indicate detection limit of 40. The ID50s below detection limit were set as 20. The error bars represent standard deviation from triplicate measurements.

Encouraged by the durable S2P-binding antibody response elicited by S6P-HBsAg, we further tested the neutralization potency of the pooled sera from each group from week 22 to week 45. At 10 µg dose, the neutralization activity was detected up to week 22 for soluble S2P and up to week 45 for soluble S6P and S6P-HBsAg at comparable levels (Figs. 2 and 4; Supplementary Figs. 8 and 11). While the neutralization activity was only detected up to week 14 for 2 and 0.4 µg soluble S2P and S6P groups, the neutralization activity was maintained at substantially higher levels through week 45 for the same dose of S6P-HBsAg groups, which lasted about 7 months longer than the duration of soluble S2P and S6P groups. The only exception was the 0.4 µg S6P-12-HBsAg group which showed no neutralization potency at week 45.

### DNA encoding SARS-CoV-2 S6P-HBsAgs elicits greater nAb responses against WA1 and B.1.1.529 pseudoviruses than DNA encoding S2P(1-1273) or soluble S6P in mice preimmunized with Recombivax HB

**Fig. 5:**
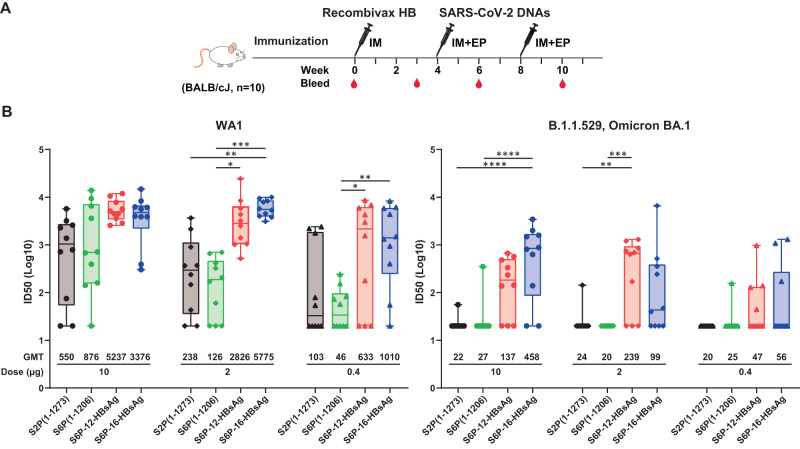
Neutralization potency elicited by SARS-CoV-2 S6P-HBsAgs in mice preimmunized with Recombivax HB. **A** Immunization schema of SARS-CoV-2 constructs. SARS-CoV-2 S2P(1-1273) DNA matching the sequence of mRNA-1273, S6P(1-1206), S6P-12-HBsAg and S6P-16-HBsAg were administered to mice preimmunized with Recombivax HB. **B** Neutralization ID50s against WA1 (left) and B.1.1.529 (BA.1, right) pseudoviruses at week 10. The ID50s were shown in a logarithmic scale in the box and whiskers format. The data points in the median quartile of each group were boxed. The error bars represent 95% confidence interval for the ID50s. The doses and immunogens were indicated. The data from each animal were shown as dots, diamonds and triangles for 10, 2, and 0.4 µg, respectively. The data points were shown in black, yellow green, red and blue for S2P(1-1273), S6P(1-1206), S6P-12-HBsAg and S6P-12-HBsAg, respectively. The geometric mean ID50 values were indicated. The statistical analyses were performed using the two-way ANOVA test following log transformation of the data. *p < 0.05; **p < 0.01; ***p < 0.001; ****p < 0.0001.

As a large population worldwide has been exposed to HBsAg by either natural infection or hepatitis B vaccination^37,38^, we tested whether SARS-CoV-2 S6P-HBsAgs can elicit potent antibody responses against SARS-CoV-2 in mice preimmunized with Recombivax HB. Recombivax HB is a vaccine against hepatitis B virus (HBV) manufactured by Merck. Two DNA immunizations were administered at weeks 4 and 8 to mice preimmunized with Recombivax HB at week 0 (Fig. 5A). Potent anti-HBsAg antibodies were detected from all groups of animals at weeks 3, 6, and 10 (Supplementary Fig. 12). Following DNA immunizations plus electroporation, 10, 2, and 0.4 µg S6P-HBsAg elicited comparable levels of anti-WA1 S2P antibodies at week 6 to those elicited by the same dose of non-nanoparticle forms of S2P(1-1273) and S6P(1-1206) (Supplementary Fig. 13). At 2 weeks post the 2nd immunization, 2 and 0.4 µg S6P-16-HBsAg elicited significantly higher levels of S2P-binding antibodies than the same dose of S2P(1-1273).

In terms of neutralizing antibody responses, we detected significantly increased WA1 ID50s and ID80s in 2 and 0.4 µg of S6P-12-HBsAg and S6P-16-HBsAg groups than in the same dose of spike groups (Fig. 5B, Supplementary Fig. 14A, Supplementary Table 7). 0.4 to 10 µg S6P-HBsAgs elicited 6.1–24.3-fold and 3.9–45.8-fold higher geometric mean ID50s than the same dose of S2P(1-1273) and soluble S6P, respectively (Supplementary Table 8). Between mice without and with Recombivax HB pre-vaccination, we observed up to 7.5-fold difference in ID50s at 2 weeks post the second DNA immunization (Supplementary Table 9). The ID80s at week 10 from mice preimmunized with Recombivax HB in the 10 and 2 µg S6P-HBsAg groups were comparable to the ID50s at week 6 in mice without Recombivax HB preimmunization but immunized with the same immunogens (Supplementary Fig. 15). Two immunizations of S6P-HBsAgs also elicited potent binding (Supplementary Fig. 13) and neutralizing antibody responses against BA.1 pseudovirus, with significantly higher ID50s and ID80s than those elicited by S2P(1-1273) and S6P(1-1206) (Fig. 5B, Supplementary Fig. 14B, Supplementary Table 8).

To test whether S6P-HBsAg can also serve as a booster immunization for HBV vaccine, we tested the neutralization potency of the mouse sera against HBV. Neutralization potency against live HBV viruses were only detected in mice preimmunized with Recombivax HB followed by two immunizations of 10 µg of SARS-CoV-2 S2P(1-1273), S6P(1-1206), S6P-12-HBsAg or S6P-16-HBsAg DNAs (Supplementary Fig. 14C). The neutralization potency appeared to be higher in 10 µg S2P(1-1273) and S6P(1-1206) groups than in 10 µg of either S6P-HBsAg group.

## Discussion

In this study, we designed and characterized self-assembling SARS-CoV-2 spike-HBsAg nanoparticles and assessed their immunogenicity in mice via genetic delivery. We show that these HBsAg nanoparticles displaying SARS-CoV-2 S6P elicit potent, broad and durable immune responses compared to non-nanoparticle form of stabilized SARS-CoV-2 spikes. Additionally, preimmunization with a HBV vaccine leads to a further enhancement of the HBsAg nanoparticle-elicited immune responses against SARS-CoV-2 and its variants.

SARS-CoV-2 spike exhibits a metastable prefusion conformation that spontaneously transitions to its post-fusion conformation^3^. The two-proline-stabilized SARS-CoV-2 spike S2P in its prefusion conformation has been used in the current COVID-19 vaccines from Moderna, Pfizer-BioNTech and Johnson & Johnson. We have screened WA1 spike, S2P and S6P for nanoparticle formation on a HBsAg core. S6P succeeded to form well-defined HBsAg nanoparticles whereas small fractions of wild type spike and S2P formed nanoparticles. This may be due to the metastability of SARS-CoV-2 spike. S2P is not as stable as S6P, as four proline substitutions in S6P in addition to the K986P and V987P substitutions further stabilize its prefusion conformation^2,8^. Both S6P-12-HBsAg and S6P-16-HBsAg bound well to human ACE2 and mAbs specific to the RBD or NTD domain of SARS-CoV-2 spike, with comparable or stronger affinity as compared with free S6P, suggesting the accessibility of the neutralizing epitopes in the S6P-HBsAg nanoparticles. The reduced binding of S6P-HBsAg shown by ELISA was likely due to less molarity of and less accessible area to the coated spike on the HBsAg core.

DNA encoding S6P-12-HBsAg and S6P-16-HBsAg elicited potent nAb responses to SARS-CoV-2 and its variants. The lower S2P-binding antibody levels but higher nAb responses elicited by 0.4 µg S6P-HBsAg than those elicited by the same dose of soluble S2P and S6P are indicative of the higher RBD-specific neutralizing antibodies elicited by S6P-HBsAg. In addition, the nAb responses elicited by S6P-HBsAg persisted through week 45 and is more durable than those elicited by soluble S2P and S6P at low doses via genetic delivery. Though both soluble S2P and S6P as well as S6P-HBsAg elicited persistent S2P-binding antibody responses, S6P-HBsAg elicited significantly higher proportions of RBD-binding antibodies while less NTD-binding antibodies than soluble S2P and S6P, which may result in a higher fraction of nAbs and neutralizing epitope specific long-lived plasma cells, leading to more potent and durable nAb responses. The presence of HBsAg in the S6P-HBsAg nanoparticles might also contribute to this durable memory, as HBV vaccines are able to elicit about 30 years of memory against HBsAg^27,28^. The detailed mechanisms underlying the durable nAb responses elicited by S6P-HBsAg remain to be addressed. Similar to our findings, persistent binding antibody responses along with waning nAb responses over time have been observed in people post mRNA-1273 vaccination or post natural infection of SARS-CoV-2^39,40^. The persistent binding antibody responses accompanied by waning nAb responses are likely due to the presence of large fraction of long-lived plasma cells elicited by non-neutralizing epitopes of SARS-CoV-2 spike, which remain to be investigated.

The neutralization potency induced by DNA delivery of S6P-HBsAgs is comparable to that elicited by intramuscular delivery of 1 µg mRNA-1273 or SARS-CoV-2 S2P protein^7,41^, albeit more uniform responses were induced by mRNA-1273. Using the same route of genetic delivery, S6P-HBsAgs elicited substantially higher neutralization potency than S2P (1-1273), whose coding sequence matches that in mRNA-1273. Similar observations were noted in mice preimmunized with a HBV vaccine, with further significantly enhanced potency. The higher neutralization potency elicited by S6P-HBsAgs than by non-nanoparticle forms of stabilized spike alone could be contributed by several factors. First, S6P-HBsAg nanoparticles with diameters of ~ 70 nm are much larger than the SARS-CoV-2 spike. The large size of these nanoparticles may result in more efficient internalization of the spike by antigen presenting cells (APCs) and retention of the spike on lymph node follicles^42^. The repetitive array of the spikes on S6P-HBsAg nanoparticles may also enable efficient binding and activation of multiple B cell receptors. In addition, the antibody responses elicited by S6P-HBsAgs were mainly directed against the spikes displayed on the HBsAg nanoparticles as opposed to HBsAg, suggesting efficient presentation of the spike on the surface of these nanoparticles by the HBsAg core. Lastly, it has been shown that the anti-HBsAg seroconversion rates in humans increase for a period up to 8 months post Recombivax HB immunizations^43^, and the GMTs persist at high levels for 13 months as tested^44^, indicative of a prolonged period of immune activation after Recombivax HB vaccination. Such prolonged immune responses elicited by Recombivax HB might have contributed to the remarkably increased neutralization potency in mice preimmunized with Recombivax HB, as compared to immunized mice originally naïve to HBsAg.

Two immunizations of S6P-HBsAgs elicited robust neutralization potency against B.1.1.529 (Omicron BA.1) pseudovirus, in mice preimmunized with Recombivax HB. While a 3rd dose of mRNA vaccines is needed to offer better protection against Omicron variants^14,15^ and a fourth dose retains low efficacy in preventing Omicron infections^16^, and the bivalent COVID-19 vaccines do not resolve the durability issue, S6P-HBsAgs may provide a strategy to offer longer and better protection against SARS-CoV-2 in populations that have previously exposed to HBsAg by infection or vaccination. As per WHO estimates, approximately 1/3 of the global population have been infected with HBV in 2010^37^. The immunization rate with 3 doses of HBV vaccine during infancy has also reached 85% worldwide in 2019^38^. This high rate of exposure to HBsAg in global population further highlights the potential of SARS-CoV-2 S6P-HBsAg as a next generation genetic vaccine platform for inducing more potent and durable protection against SARS-CoV-2.

Many nanoparticle designs require an additional protein conjugation step^18–20,25,26^, which does not allow them to be amenable to gene-based delivery. Our S6P-HBsAg designs exhibit advantages as genetic vaccine candidates over similar nanoparticle vaccine designs utilizing an additional conjugation step^18,19,25^. The utilization of spike in our designs enables more neutralizing epitopes than RBD-based vaccine designs, as NTD and S2 are known to be able to elicit neutralizing antibodies against SARS-CoV-2 (Supplementary Table 1, references attached). The preexisting immunological memory against HBsAg in large population globally may also enable S6P-HBsAgs to leverage their potency for vaccine development against SAR-CoV-2 and other related coronaviruses. Our DNA constructs encode the HBsAg-spike as a single gene product and could readily be encoded by mRNA or viral vector gene delivery vaccine platform.

One limitation of this study is that it was done with DNA, which is highly immunogenic in mice, but less immunogenic than mRNA in nonhuman primates (NHP) and humans. Therefore, further studies should evaluate mRNAs encoding these S6P-HBsAg particles in NHP and investigate the mechanism underlying the enhanced vaccine-induced immune response by a HBV pre-vaccination.

In conclusion, SARS-CoV-2 S6P-HBsAg can elicit more potent and durable nAb responses against diverse SARS-CoV-2 strains than non-nanoparticle form of stabilized spikes, including SARS-CoV-2 full-length S2P with its coding sequence matching that in mRNA-1273. The nAb responses elicited by S6P-HBsAg can persist 7 months longer than soluble stabilized spikes and appeared to be enhanced by pre-exposure to HBsAg. S6P-HBsAgs represent promising next generation genetic vaccine candidates against SARS-CoV-2. Overall, this platform has the potential to serve as a universal vaccine platform against coronaviruses and other infectious pathogens.

## Methods

### DNA construct design

The HBsAg nanoparticles displaying SARS-CoV-2 spike, S2P or S6P were designed as spike-HBsAg fusion proteins. The coding sequence of the ectodomain (aa 1-1206) of SARS-CoV-2 WA1 spike (NC_045512.2), 2P- or 6P-stabilized mutant (S2P or S6P)^2,8^ was joined with the coding sequence of HBsAg (aa 1-226, AET06188.1). The linker was made up of varying repeats of GS dipeptide. The coding sequences of SARS-CoV-2 spike-HBsAgs were inserted into the CMVR8400 vector via XbaI and BamHI sites. The ectodomain of SARS-CoV-2 S2P or S6P, as well as the full-length S2P (aa 1-1273) were also constructed in the CMVR8400 vector via the same restriction sites as above, respectively. Each construct was human codon-optimized, synthesized by GenScript and confirmed by DNA sequencing.

### Protein expression and purification

The SARS-CoV-2 spike-HBsAg plasmids were transfected into Expi293F cells using ExpiFectamine 293 transfection kit following the manufacturer’s instructions. The transfected culture was grown at 37 °C for 5 days before harvest. The culture was then spun down at 10,000 × *g* at 20 °C for 30 min. The supernatant was collected and passed through a 0.2 µm filter. The supernatant was further spun through 20% sucrose cushion in a buffer containing 20 mM MES, 150 mM NaCl, pH 6.0. The ultracentrifugation was performed with a Surespin rotor at 71552 × g, 4 °C for 2 h. The pellet was resuspended in a buffer containing 20 mM MES, 150 mM NaCl, pH 6.0, filtered through a 0.45 µm filter, and then spun through a sucrose gradient consisting of 1.5 ml of 20% to 65% sucrose in a buffer containing 20 mM MES, 150 mM NaCl, pH 6.0. This step of ultracentrifugation was done with a Th-641 rotor at 217,339 × *g*, 4 °C for 8 h. The sucrose fractions were collected and stored at 4 °C. A dialysis against PBS buffer, pH 7.4, was performed before use. SARS-CoV-2 WA1 S2P and S6P expression plasmids were provided by Dr. Barney Graham and Professor Jason McLellan^2,8^. B.1.351, B.1.617.2 and B.1.1.529 S2Ps were constructed in the CMVR8400 vector via XbaI and BamHI sites using the same cloning strategy. The coding sequences were human codon optimized and confirmed by DNA sequencing. The stabilized WA1 and variant spikes were expressed following the same protocol and purified using Complete His-Tag Purification Resin (Roche, for WA1 S2P and S6P) or StrepTactin resin (IBA, for variant S2Ps). The tags in S2P and S6P were cleaved with HRV-3C and further purified by sizing column purification in PBS. Fc-tagged human ACE2, His-tagged SARS-CoV-2 RBD, NTD recombinant proteins were expressed likewise and purified using rProtA Sepharose and Ni-NTA resin, respectively, before sizing column purification^7,45^. The purified proteins were aliquoted, frozen in liquid nitrogen, and kept at −80 °C before use.

### Negative-stain electron microscopy

The sucrose fractions of SARS-CoV-2 spike-HBsAg fusion proteins were buffer exchanged to PBS containing 5–10% sucrose, and then applied to a freshly glow-discharged carbon-coated grid for about 15 s. The grid was washed with buffer containing 10 mM HEPES, pH 7.0, and 150 mM NaCl, followed by negative staining with 0.7% uranyl formate. Images were collected at a nominal magnification of 57,000 using EPU software on a Thermo Scientific Talos F200C electron microscope operated at 200 kV and equipped with a 4k × 4k Ceta CCD camera or at a nominal magnification of 50,000 using SerialEM^46^ on an FEI T20 electron microscope operated at 200 kV and equipped with an Eagle CCD camera. The corresponding pixel sizes were 0.253 and 0.22 nm. Particles were picked using e2boxer from the EMAN2 software package^47^. Reference-free 2D classification was performed using Relion^48^.

### ELISA to assess ACE2 binding and antigenicity of SARS-CoV-2 spike-HBsAgs

ELISA plates (Thermo Fisher, 442404) were coated with 1 µg/ml of SARS-CoV-2 WA1 or variant S2P, WA1 S6P or S6P-HBsAg in PBS buffer, pH 7.4, 100 µl/well at 4 °C for 16 h^2^. The plates were washed with PBST thrice, 300 µl per well per time and then blocked with 5% skim milk in 1× PBST at room temperature for 1 h. The binding of SARS-CoV-2 WA1 S2P, S6P and spike-HBsAg was done with 4 µg/ml SARS-CoV-2 mAbs (Supplementary Table 1), followed by 7 data points of 4-fold serial dilutions or with 100 µg/ml human ACE2, followed by 11 data points of 4-fold serial dilutions^49^. The goat anti-human IgG Fc-HRP antibody (Invitrogen, A18817, 1/5000) was used to detect the ACE2 binding. The primary antibody incubation was done at room temperature for 30 min. Following three washes, the plates were incubated with HRP-conjugated anti-human or anti-mouse antibody (Thermo Fisher, A18811 and G21040, 1/2000) at room temperature for 30 min. The plates were then washed thrice and developed with 3,5,3′,5′-tetramethylbenzidine (TMB) (KPL) at room temperature for 10 min. After quenched with 1 N H_2_SO_4_ (Fisher), the plates were read at 450 nm with a SpectraMax Plus 384 microplate reader. The data were plotted and analyzed with GraphPad Prism.

### Bio-layer interferometry (BLI)

The BLI binding of SARS-CoV-2 stabilized spike and HBsAg nanoparticles to SARS-CoV-2 mAbs or human ACE2 was measured by an Octet HTX instrument. The binding buffer contained 1x HBS-EP+ buffer (GE) and 5% sucrose. The mAbs or human ACE2 with a Fc tag were captured by AHC or AMC sensors to yield a binding signal of 1–1.3 nm. The SARS-CoV-2 stabilized spike free or displayed on a HBsAg core varied from 0 to 800 nM. The molar concentration of the S6P-HBsAg was determined based on spike (but not based on a nanoparticle). The association and dissociation were monitored for 300 s at 30 °C, respectively. The binding curves were globally fitted with a 1:1 Langmuir binding model using Data Analysis Software v9.0.

### Immunogenicity evaluation in mice

The immunogenicity studies in 6- to 8-week-old female BALB/cJ mice (Jackson Laboratory) were performed in compliance with all pertinent US NIH regulations and approval from the Animal Care and Use Committee (ACUC) of the Vaccine Research Center (VRC). Pre-bleeds were collected the day before the first immunization. Plasmid DNAs encoding SARS-CoV-2 WA1 S2P(1-1206), S6P(1-1206), S6P-12-HBsAg, S6P-16-HBsAg, S2P(1-1273) were diluted prior to immunization in PBS, pH 7.4. Naïve mice were injected intramuscularly twice spaced 4-week apart in both hind legs with a total of 10, 2 or 0.4 µg plasmid DNA in 100 µl PBS, pH 7.4. S2P(1-1273) was administered twice with a 3-week interval to match the immunization regimen of BNT162b2. Mice preimmunized with 1 µg Recombivax HB vaccine intramuscularly^50^ at week 0 received two immunizations of 10, 2 or 0.4 µg SARS-CoV-2 WA1 S2P(1-1273), S6P(1-1206), S6P-12-HBsAg and S6P-16-HBsAg DNA at week 4 and week 8, respectively. Electroporation was done following each DNA immunization with the AgilePulse System (Harvard Apparatus) using manufacturer-recommended setting^51^. The electroporation was applied to the muscles at the injection sites in both hind legs. Mouse sera were collected 2 weeks post each DNA immunization, and 3 weeks post Recombivax HB vaccination^50^.

### Serum ELISA

ELISA plates (Thermo Fisher, 442404) were coated with 1 µg/ml of SARS-CoV-2 WA1, variant S2P, or HBsAg (ProspecBio, HBS-875) in PBS, pH 7.4 at 4 °C for 16 h^7^. Standard washes and blocking steps were done as described above. The plates were blocked for 2 h. The sera were diluted by 100-fold in 5% skim milk in PBST. A serial 4-fold dilution for weeks 0 and 2 sera or 6-fold dilution for week 6 sera was applied to the 100-fold dilution preparations. The plates were incubated with diluted sera at room temperature for 1 h. The HRP-conjugated anti-mouse secondary antibody (Thermo Fisher, G21040, 1/2000) was used to detect the antibody responses. The endpoint titers were calculated as the dilution that yielded an optical density equivalent to 4×background (secondary antibody alone).

### Pseudovirus neutralization assay

The codon-optimized SARS-CoV-2 spike (Wuhan-1, GenBank: MN908947.3; D614G, B.1.351, B.1.617.2, B.1.1.529) plasmids were used^30^. Pseudoviruses were generated by co-transfection of transducing plasmid pHR’ CMV-Luc encoding a luciferase reporter, lentivirus packaging plasmid pCMVd8.2, a TMPRSS2 plasmid and a spike plasmid of SARS-CoV-2 or variants into HEK293T/17 cells (ATCC CRL-11268) using Lipofectamine 3000 transfection reagent (Thermo Fisher, L3000-001)^7,30,52^. Heat-inactivated serum was mixed with the titrated pseudoviruses, incubated, and then added to pre-plated 293T-ACE2 cells (from Dr. Michael Farzan) in triplicate. Following 2 h of incubation, wells were replenished with 150 µl of fresh media. 72 h later, the cells were lysed and the luciferase activity was recorded in relative light units (RLU). The neutralization activity was normalized to uninfected cells as 100% neutralization and to cells infected with only pseudovirus as 0% neutralization. ID50 and ID80 titers were determined using a log (agonist) vs normalized response (variable slope) nonlinear function in GraphPad Prism.

### HBV neutralization assay

Group pooled sera from mice immunized with 10 µg of SARS-CoV-2 DNAs were tested for neutralization potency against live HBV viruses (subtype *ayw*, ImQuest BioSciences). Week 22 sera from mice without Recombivax HB preimmunization and week 10 sera from mice with Recombivax HB preimmunization were evaluated. HepG2-NTCP cells (Baruch S. Blumberg Institute) were seeded in a 48-well plate and incubated for 24 h in DMEM F12 (Gibco, 11320-030) supplemented with 10% FBS, 1% Fungizone and 2 µg/mL Puromycin. Pooled sera were diluted in the medium with a 4-fold series of 7 dilutions. The diluted sera were incubated with HBV viruses (MOI: 1000) for 45 min at 37 °C with 5% CO_2_. Following the incubation, media was removed from the pre-seeded plates. The serum/virus samples were added in triplicate to the cells and incubated for 18 to 24 h. A neutralizing mAb against HBV H015 (Acrobiosystems, HBG-M406-50ug) up to 1 µg/ml and Tenofovir disoproxil fumarate (TDF, Gilead) up to 1 µM were used as positive controls. The virus and compound were then removed by 5 washes and replaced with fresh media with and without H015 or TDF. This was repeated on day 4 and day 7 post infection. On day 10 the supernatant was collected and evaluated by qPCR assay. The primers and probe were from IDT and listed as follows:

HBV-AD38-qF1: 5′-CCGTCTGTGCCTTCTCAT CTG-3′; HBV-AD38-qR1: 5′-AGTCCAAGAGTCCTCTTATACAAGACC-3′; and HBV-AD38-qP1: 5′-FAM/CCGTGTGCA/ZEN/CTTCGCTTCAC-3′ BHQ1.

### Reporting summary

Further information on research design is available in the Nature Research Reporting Summary linked to this article.

### Supplementary information

Supplementary material

REPORTING SUMMARY

## Data Availability

All data are available in the manuscript or the supplementary material. Correspondence and requests for materials should be addressed to A.P. (pegua@niaid.nih.gov).
