# Supplementary material for: Self-assembling SARS-CoV-2 spike-HBsAg nanoparticles elicit potent and durable neutralizing antibody responses via genetic delivery
Source: NPJ Vaccines. 2023 Aug 8;8:111. doi: 10.1038/s41541-023-00707-w (PMC10409857; doi:10.1038/s41541-023-00707-w)
Supplement: Supplementary file 1 — Supplementary material [file 41541_2023_707_MOESM1_ESM.pdf]

Supplementary materials for

Self-Assembling SARS-CoV-2 Spike-HBsAg Nanoparticles Elicit Potent and Durable Neutralizing Antibody Responses via Genetic Delivery

Cuiping Liu *et al*.

List of Supplementary Tables

Supplementary Table 1. mAbs used in this study to evaluate the antigenicity of SARS-CoV-2 S6P-HBsAg nanoparticles

Supplementary Table 2. Binding affinity of SARS-CoV-2 S6P-HBsAg nanoparticles to SARS-CoV-2 mAbs and human ACE2 by Bio-Layer Interferometry (BLI) (to Fig. S4)

Supplementary Table 3. Geometric mean neutralization ID50 titers against WA1 pseudovirus at week 6 (to Fig. 2B)

Supplementary Table 4. Statistical analyses of geometric mean ID50s at week 6 against WA1 pseudovirus (to Fig. 2B)

Supplementary Table 5. Geometric mean neutralization ID50 titers against SARS-CoV-2 variant pseudovirus at week 6 (to Fig. 3)

Supplementary Table 6. Statistical analyses of geometric mean ID50s at week 6 against SARS-CoV-2 variant pseudoviruses (to Fig. 3)

Supplementary Table 7. Statistical analyses of geometric mean ID50s at week 10 for mice preimmunized with Recombivax HB (to Fig. 5)

Supplementary Table 8. Geometric mean neutralization ID50 titers at week 10 in mice preimmunized with Recombivax HB (to Fig. 5)

Supplementary Table 9. Comparison of ID50s between mice with and without RecombiVax HB preimmunization

Supplementary Table 1. mAbs used in this study to evaluate the antigenicity of SARS-CoV-2 S6P-HBsAg nanoparticles

Antibody	Targeting domain of SARS-CoV-2 spike	Class of RBD mAbs	Reference
4A8	NTD		[1]
S652-118	NTD		[2]
4-19	NTD		[3]
4-8	NTD		[3]
1-68	NTD		[3]
A20-36.1	SD1		[4]
B1-182.1	RBD up	Class I	[5]
S2E12	RBD up	Class I	[6]
REGN10933	RBD up	Class I	[7, 8]
CB6	RBD up	Class I	[9]
A23-58.1	RBD up	Class I	[5]
A19-46.1	RBD	Class II	[5, 10]
LY-CoV555	RBD	Class II	[11, 12]
LY-CoV1404	RBD	Class III	[13]
A19-61.1	RBD	Class III	[5]
REGN10987	RBD	Class III	[7, 8]
S309	RBD	Class III	[14]
ADG2	RBD	Class I/IV	[10, 15, 16]
DH1047	RBD	Class I/IV	[16, 17]
S2H97	RBD	Class V	[6]
S2P6	S2		[18]
S652-112	S2		[2]
WS6	S2		[19]
CoVA1-07	S2		[20]
CoVA2-14	S2		[20]
CoVA2-18	S2		[20]

Supplementary Table 2. Binding affinity of SARS-CoV-2 S6P-HBsAg nanoparticles to SARS-CoV-2 mAbs and human ACE2 by Bio-Layer Interferometry (BLI) (to Fig. S4)

	K_d (×10^{-7} M)					
	**4A8**	**S652-118**	**4-19**	**4-8**	**1-68**	**A20-36.1**
S2P	1.0 ± 0.1	0.9 ± 0.2	2.5 ± 0.3	2.8 ± 0.4	5.6 ± 1.1	0.3 ± 0.0
S6P	1.6 ± 0.1	10.0 ± 6.0	7.9 ± 3.0	4.6 ± 0.4	4.8 ± 2.5	4.5 ± 0.7
S6P-8-HBsAg	0.9 ± 0.3	0.5 ± 0.1	2.7 ± 0.9	0.9 ± 0.2	7.6 ± 3.9	1.1 ± 0.2
S6P-12-HBsAg	2.1 ± 0.5	1.3 ± 0.2	1.0 ± 0.2	2.7 ± 0.3	0.9 ± 0.1	0.9 ± 0.1
S6P-16-HBsAg	0.8 ± 0.1	0.8 ± 0.1	0.4 ± 0.1	1.2 ± 0.2	0.6 ± 0.1	0.5 ± 0.1
	**S2P6**	**S652-112**	**WS6**	**CoVA1-07**	**CoVA2-14**	**CoVA2-18**
S2P	0.7 ± 0.1	1.9 ± 0.3	3.6 ± 0.9	0.7 ± 0.1	1.3 ± 0.2	5.3 ± 0.5
S6P	3.6 ± 0.4	8.9 ± 1.5	3.5 ± 0.1	7.6 ± 0.5	8.4 ± 0.9	40.0 ± 5.0
S6P-8-HBsAg	1.4 ± 0.4	2.6 ± 1.9	4.7 ± 1.8	NB	NB	NB
S6P-12-HBsAg	1.5 ± 0.8	1.4 ± 0.4	1.9 ± 0.3	NB	NB	NB
S6P-16-HBsAg	0.4 ± 0.1	0.5 ± 0.1	1.6 ± 0.6	NB	NB	NB
S2				0.3 ± 0.0	0.4 ± 0.0	2.7 ± 0.3
	**B1-182.1**	**S2E12**	**REGN10933**	**CB6**	**A23-58.1**	**A19-46.1**
S2P	0.2 ± 0.0	0.2 ± 0.0	0.2 ± 0.1	0.4 ± 0.1	0.4 ± 0.1	0.3 ± 0.0
S6P	0.9 ± 0.1	1.4 ± 0.2	0.8 ± 0.5	2.3 ± 0.3	1.7 ± 0.4	2.7 ± 0.4
S6P-8-HBsAg	0.4 ± 0.1	1.5 ± 0.4	1.4 ± 0.2	2.6 ± 0.4	1.2 ± 0.2	0.2 ± 0.0
S6P-12-HBsAg	0.5 ± 0.1	1.4 ± 0.5	1.0 ± 0.3	2.1 ± 0.3	0.8 ± 0.2	2.7 ± 0.8
S6P-16-HBsAg	1.5 ± 0.4	0.9 ± 0.1	0.4 ± 0.0	0.6 ± 0.2	0.2 ± 0.1	0.3 ± 0.1
	**LY-CoV555**	**LY-CoV1404**	**A19-61.1**	**REGN10987**	**S309**	**ADG2**
S2P	0.2 ± 0.0	0.5 ± 0.1	1.0 ± 0.1	0.2 ± 0.0	0.8 ± 0.1	0.4 ± 0.0
S6P	0.9 ± 0.1	1.4 ± 0.2	2.9 ± 0.5	0.8 ± 0.1	4.2 ± 1.9	1.6 ± 0.2
S6P-8-HBsAg	0.5 ± 0.2	0.7 ± 0.1	0.8 ± 0.2	1.3 ± 0.1	0.2 ± 0.1	1.2 ± 0.4
S6P-12-HBsAg	0.3 ± 0.1	1.1 ± 0.4	0.5 ± 0.0	1.1 ± 0.2	0.5 ± 0.1	4.3 ± 2.0
S6P-16-HBsAg	0.2 ± 0.0	0.3 ± 0.1	0.3 ± 0.1	0.4 ± 0.1	0.5 ± 0.1	2.0 ± 0.7
	**S2H97**	**DH1047**	**Human ACE2**			
S2P	0.2 ± 0.0	0.7 ± 0.1	0.5 ± 0.1			
S6P	1.8 ± 0.4	2.3 ± 0.5	3.7 ± 0.9			
S6P-8-HBsAg	2.1 ± 0.7	17.0 ± 9.0	0.4 ± 0.0			
S6P-12-HBsAg	0.6 ± 0.1	4.7 ± 0.7	3.8 ± 1.5			
S6P-16-HBsAg	0.5 ± 0.2	1.2 ± 0.2	6.7 ± 3.2			
Note: The molar concentration of the S6P-HBsAg nanoparticles were determined based on spike but not based on nanoparticles. NB: No binding.						

Supplementary Table 3. Geometric mean neutralization ID50 titers against WA1 pseudovirus at week 6 (to Fig. 2B)

Immunogen	S2P (1-1206)	S6P (1-1206)	S2P (1-1273)	S6P-12-HBsAg	S6P-16-HBsAg	S2P (1-1206)	S6P (1-1206)	S2P (1-1273)	S6P-12-HBsAg	S6P-16-HBsAg	S2P (1-1206)	S6P (1-1206)	S2P (1-1273)	S6P-12-HBsAg	S6P-16-HBsAg
Dose	10 μg					2 μg					0.4 μg				
GMT	207	181	145	892	958	138	53	256	1406	771	70	61	87	503	374
Fold of Change in GMT	1.0	0.9	0.7	4.3	4.6	1.0	0.4	1.9	10.2	5.6	1.0	0.9	1.2	7.2	5.3
	1.1	1.0	0.8	4.9	5.3	2.6	1.0	4.8	26.5	14.5	1.1	1.0	1.4	8.2	6.1
	1.4	1.2	1.0	6.2	6.6	0.5	0.2	1.0	5.5	3.0	0.8	0.7	1.0	5.8	4.3
Overall Fold of Change in GMT				4.3~6.2	4.6~6.6				5.5~26.5	3~14.5				5.8~8.2	4.3~6.1

GMT: Geometric mean titer. Fold of change was calculated in comparison with the same dose of non-nanoparticle form of spike (highlighted in grey). The GMT and fold of change were shown in red and blue for S6P-12-HBsAg and S6P-16-HBsAg, respectively.

Supplementary Table 4. Statistical analyses of geometric mean ID50s at week 6 against WA1 pseudovirus (to Fig. 2B)

Nanoparticle	Non-nanoparticle spike	*p* value (ANOVA)
2 µg S6P-12-HBsAg	10 µg S6P(1-1206)	0.0408
	2 µg S2P(1-1206)	0.0103
	2 µg S6P(1-1206)	< 0.0001
0.4 µg S6P-12-HBsAg	2 µg S6P(1-1206)	0.0144
	0.4 µg S6P(1-1206)	0.0296
2 µg S6P-16-HBsAg	2 µg S6P(1-1206)	0.0012
10 µg S6P-12-HBsAg	10 µg S2P(1-1273)	0.043*
2 µg S6P-12-HBsAg	2 µg S2P(1-1273)	0.0345*
0.4 µg S6P-12-HBsAg	0.4 µg S2P(1-1273)	0.0341*
10 µg S6P-16-HBsAg	10 µg S2P(1-1273)	0.0286*
Note: The statistical analyses were performed using the Two-way ANOVA test for the comparison between S6P-HBsAg and soluble S2P(1-1206), S6P(1-1206). The comparison between the same dose of S6P-HBsAg and S2P(1-1273) was and done using two tailed Mann-Whitney test with Dunn’s multiple comparisons test.		

Supplementary Table 5. Geometric mean neutralization ID50 titers against SARS-CoV-2 variant pseudovirus at week 6 (to Fig. 3)

	Immunogen	S2P (1-1206)	S6P (1-1206)	S6P-12-HBsAg	S6P-16-HBsAg	S2P (1-1206)	S6P (1-1206)	S6P-12-HBsAg	S6P-16-HBsAg	S2P (1-1206)	S6P (1-1206)	S6P-12-HBsAg	S6P-16-HBsAg
	Dose	10 μg				2 μg				0.4 μg			
D614G	GMT	897	965	3444	3122	453	218	3565	2513	268	304	791	722
	Fold of Change in GMT	1.0	1.1	3.8	3.5	1.0	0.5	7.9	5.5	1.0	1.1	3.0	2.7
		0.9	1.0	3.6	3.2	2.1	1.0	16.4	11.5	0.9	1.0	2.6	2.4
	Overall Fold of Change in GMT			3.6~3.8	3.2~3.5			7.9~16.4	5.5~11.5			2.6~3.0	2.4~2.7
B.1.351	GMT	60	128	336	487	60	86	400	101	35	27	51	85
	Fold of Change in GMT	1.0	2.1	5.6	8.1	1.0	1.4	6.7	1.7	1.0	0.8	1.5	2.4
		0.5	1.0	2.6	3.8	0.7	1.0	4.7	1.2	1.3	1.0	1.9	3.1
	Overall Fold of Change in GMT			2.6~5.6	3.8~8.1			4.7~6.7	1.2~1.7			1.5~1.9	2.4~3.1
B.1.617.2	GMT	44	56	319	399	36	34	276	353	47	52	148	68
	Fold of Change in GMT	1.0	1.3	7.3	9.1	1.0	0.9	7.7	9.8	1.0	1.1	3.1	1.4
		0.8	1.0	5.7	7.1	1.1	1.0	8.1	10.4	0.9	1.0	2.8	1.3
	Overall Fold of Change in GMT			5.7~7.3	7.1 ~ 9.1			7.7 ~ 8.1	9.8~10.4			2.8~3.1	1.3~1.4
B.1.1.529 (BA.1)	GMT	42	49	164	138	37	35	23	62	24	20	44	28
	Fold of Change in GMT	1.0	1.2	3.9	3.3	1.0	0.9	0.6	1.7	1.0	0.8	1.8	1.2
		0.9	1.0	3.3	2.8	1.1	1.0	0.7	1.8	1.2	1.0	2.2	1.4
	Overall Fold of Change in GMT			3.3~3.9	2.8~3.3			0.6~0.7	1.7~1.8			1.8~2.2	1.2~1.4

GMT: Geometric mean titer. Fold of change was calculated in comparison with the ID50 against the same pseudovirus elicited by the same dose of non-nanoparticle form of spike (highlighted in grey). The GMT and fold of change were shown in red and blue for S6P-12-HBsAg and S6P-16-HBsAg, respectively.

Supplementary Table 6. Statistical analyses of geometric mean ID50s at week 6 against SARS-CoV-2 variant pseudoviruses (to Fig. 3)

Nanoparticle	Non-nanoparticle spike	Pseudovirus	*p* value
2 µg S6P-12-HBsAg	2 µg S6P(1-1206)	D614G	0.0013
2 µg S6P-16-HBsAg	2 µg S6P(1-1206)		0.0095
2 µg S6P-12-HBsAg	2 µg S2P(1-1206)	B.1.617.2	0.0383
	2 µg S6P(1-1206)		0.0312
10 µg S6P-16-HBsAg	10 µg S2P(1-1206)		0.0172
2 µg S6P-16-HBsAg	10 µg S2P(1-1206)		0.0324
	2 µg S2P(1-1206)		0.0106
	2 µg S6P(1-1206)		0.0084
Note: The statistical analyses were performed using the two-way ANOVA test.			

Supplementary Table 7. Statistical analyses of geometric mean ID50s at week 10 for mice preimmunized with Recombivax HB (to Fig. 5)

Nanoparticle	Non-nanoparticle spike	Pseudovirus	*p* value
2 µg S6P-12-HBsAg	2 µg S6P(1-1206)	WA1	0.0054
0.4 µg S6P-12-HBsAg	0.4 µg S6P(1-1206)		0.0405
2 µg S6P-16-HBsAg	2 µg S2P(1-1273)		0.0037
	2 µg S6P(1-1206)		0.0002
0.4 µg S6P-16-HBsAg	0.4 µg S6P(1-1206)		0.0059
2 µg S6P-12-HBsAg	10 µg S2P(1-1273)	Omicron BA.1	0.0036
	2 µg S2P(1-1273)		0.0034
	10 µg S6P(1-1206)		0.006
	2 µg S6P(1-1206)		0.0009
10 µg S6P-16-HBsAg	10 µg S2P(1-1273)		<0.0001
	10 µg S6P(1-1206)		<0.0001
Note: The statistical analyses were performed using the Two-way ANOVA test.			

Supplementary Table 8. Geometric mean neutralization ID50 titers at week 10 in mice preimmunized with Recombivax HB (to Fig. 5)

	Immunogen	S2P (1-1273)	S6P (1-1206)	S6P-12-HBsAg	S6P-16-HBsAg	S2P (1-1273)	S6P (1-1206)	S6P-12-HBsAg	S6P-16-HBsAg	S2P (1-1273)	S6P (1-1206)	S6P-12-HBsAg	S6P-16-HBsAg
	Dose	10 µg				2 µg				0.4 µg			
WA1	GMT	550	876	5237	3376	238	126	2826	5775	103	46	633	1010
	Fold of Change in GMT	1.0	1.6	9.5	6.1	1.0	0.5	11.9	24.3	1.0	0.4	6.1	9.8
		0.6	1.0	6.0	3.9	1.9	1.0	22.4	45.8	2.2	1.0	13.8	22.0
	Overall Fold of Change in GMT			6.0~9.5	3.9~6.1			11.9~22.4	24.3~45.8			6.1~13.8	9.8~22.0
B.1.1.529 (BA.1)	GMT	22	27	137	458	24	20	239	99	20	25	47	56
	Fold of Change in GMT	1.0	1.2	6.2	20.8	1.0	0.8	10.0	4.1	1.0	1.3	2.4	2.8
		0.8	1.0	5.1	17.0	1.2	1.0	12.0	5.0	0.8	1.0	1.9	2.2
	Overall Fold of Change in GMT			5.1~6.2	17.0~20.8			10.0~12.0	4.1~5.0			1.9~2.4	2.2~2.8

GMT: Geometric mean titer. Fold of change was calculated in comparison with the ID50 against the same pseudovirus elicited by the same dose of non-nanoparticle form of spike (highlighted in grey). The GMT and fold of change were shown in red and blue for S6P-12-HBsAg and S6P-16-HBsAg, respectively.

Supplementary Table 9. Comparison of ID50s between mice with and without RecombiVax HB preimmunization

Prime & boost immunogen	Dose (μg)	Geometric mean ID50 against WA1			Geometric mean ID50 against BA.1		
				Ratio			Ratio
		RecombiVax HB – Week 6	RecombiVax HB + Week 10	$\frac{\text{RecombiVax HB} +}{\text{RecombiVax HB} -}$	RecombiVax HB – Week 6	RecombiVax HB + Week 10	$\frac{\text{RecombiVax HB} +}{\text{RecombiVax HB} -}$
S6P(1-1206)	10	181	876	4.8	49	27	0.6
	2	53	126	2.4	35	20	0.6
	0.4	61	46	0.8	20	25	1.3
S6P-12-HBsAg	10	892	5237	5.9	164	137	0.8
	2	1406	2826	2.0	23	239	10.4
	0.4	503	633	1.3	44	47	1.1
S6P-16-HBsAg	10	958	3376	3.5	138	458	3.3
	2	771	5775	7.5	62	99	1.6
	0.4	374	1010	2.7	28	56	2.0
S2P(1-1273)	10	145	550	3.8	20	22	1.1
	2	256	238	0.9	21.8	24	1.1
	0.4	87	103	1.2	20	20	1.0

References

1. Chi X, Y.R., Zhang J, Zhang G, Zhang Y, Hao M, Zhang Z, Fan P, Dong Y, Yang Y, Chen Z, Guo Y, Zhang J, Li Y, Song X, Chen Y, Xia L, Fu L, Hou L, Xu J, Yu C, Li J, Zhou Q, Chen W, *A neutralizing human antibody binds to the N-terminal domain of the Spike protein of SARS-CoV-2.* Science, 2020. **369**(6504): p. 6.
2. Zhou T, T.I., Olia AS, Cerutti G, Gorman J, Nazzari A, Shi W, Tsybovsky Y, Wang L, Wang S, Zhang B, Zhang Y, Katsamba PS, Petrova Y, Banach BB, Fahad AS, Liu L, Lopez Acevedo SN, Madan B, Oliveira de Souza M, Pan X, Wang P, Wolfe JR, Yin M, Ho DD, Phung E, DiPiazza A, Chang LA, Abiona OM, Corbett KS, DeKosky BJ, Graham BS, Mascola JR, Misasi J, Ruckwardt T, Sullivan NJ, Shapiro L, Kwong PD, *Structure-Based Design with Tag-Based Purification and In-Process Biotinylation Enable Streamlined Development of SARS-CoV-2 Spike Molecular Probes.* Cell Rep, 2020. **33**(4).
3. Liu L, W.P., Nair MS, Yu J, Rapp M, Wang Q, Luo Y, Chan JF, Sahi V, Figueroa A, Guo XV, Cerutti G, Bimela J, Gorman J, Zhou T, Chen Z, Yuen KY, Kwong PD, Sodroski JG, Yin MT, Sheng Z, Huang Y, Shapiro L, Ho DD, *Potent neutralizing antibodies against multiple epitopes on SARS-CoV-2 spike.* Nature, 2020. **584**(7821): p. 7.
4. Corbett KS, G.M., Wagner DA, O' Connell S, Narpala SR, Flebbe DR, Andrew SF, Davis RL, Flynn B, Johnston TS, Stringham CD, Lai L, Valentin D, Van Ry A, Flinchbaugh Z, Werner AP, Moliva JI, Sriparna M, O'Dell S, Schmidt SD, Tucker C, Choi A, Koch M, Bock KW, Minai M, Nagata BM, Alvarado GS, Henry AR, Laboune F, Schramm CA, Zhang Y, Yang ES, Wang L, Choe M, Boyoglu-Barnum S, Wei S, Lamb E, Nurmukhambetova ST, Provost SJ, Donaldson MM, Marquez J, Todd JM, Cook A, Dodson A, Pekosz A, Boritz E, Ploquin A, Doria-Rose N, Pessaint L, Andersen H, Foulds KE, Misasi J, Wu K, Carfi A, Nason MC, Mascola J, Moore IN, Edwards DK, Lewis MG, Suthar MS, Roederer M, McDermott A, Douek DC, Sullivan NJ, Graham BS, Seder RA., *Protection against SARS-CoV-2 Beta variant in mRNA-1273 vaccine-boosted nonhuman primates.* Science, 2021. **374**(6573): p. 11.
5. Wang L, Z.T., Zhang Y, Yang ES, Schramm CA, Shi W, Pegu A, Oloniniyi OK, Henry AR, Darko S, Narpala SR, Hatcher C, Martinez DR, Tsybovsky Y, Phung E, Abiona OM, Antia A, Cale EM, Chang LA, Choe M, Corbett KS, Davis RL, DiPiazza AT, Gordon IJ, Hait SH, Hermanus T, Kgagudi P, Laboune F, Leung K, Liu T, Mason RD, Nazzari AF, Novik L, O'Connell S, O'Dell S, Olia AS, Schmidt SD, Stephens T, Stringham CD, Talana CA, Teng IT, Wagner DA, Widge AT, Zhang B, Roederer M, Ledgerwood JE, Ruckwardt TJ, Gaudinski MR, Moore PL, Doria-Rose NA, Baric RS, Graham BS, McDermott AB, Douek DC, Kwong PD, Mascola JR, Sullivan NJ, Misasi J., *Ultrapotent antibodies against diverse and highly transmissible SARS-CoV-2 variants.* Science, 2021. **373**(6556).
6. Tortorici MA, B.M., Lempp FA, Pinto D, Dang HV, Rosen LE, McCallum M, Bowen J, Minola A, Jaconi S, Zatta F, De Marco A, Guarino B, Bianchi S, Lauron EJ, Tucker H, Zhou J, Peter A, Havenar-Daughton C, Wojcechowskyj JA, Case JB, Chen RE, Kaiser H, Montiel-Ruiz M, Meury M, Czudnochowski N, Spreafico R, Dillen J, Ng C, Sprugasci N, Culap K, Benigni F, Abdelnabi R, Foo SC, Schmid MA, Cameroni E, Riva A, Gabrieli A, Galli M, Pizzuto MS, Neyts J, Diamond MS, Virgin HW, Snell G, Corti D, Fink K, Veesler D., *Ultrapotent human antibodies protect against SARS-CoV-2 challenge via multiple mechanisms.* Science, 2020. **370**(6519): p. 8.
7. Baum A, A.D., Copin R, Zhou A, Lanza K, Negron N, Ni M, Wei Y, Mohammadi K, Musser B, Atwal GS, Oyejide A, Goez-Gazi Y, Dutton J, Clemmons E, Staples HM, Bartley C, Klaffke B, Alfson K, Gazi M, Gonzalez O, Dick E Jr, Carrion R Jr, Pessaint L, Porto M, Cook A, Brown R, Ali V, Greenhouse J, Taylor T, Andersen H, Lewis MG, Stahl N, Murphy AJ, Yancopoulos GD, Kyratsous

CA., *REGN-COV2 antibodies prevent and treat SARS-CoV-2 infection in rhesus macaques and hamsters.* Science, 2020. **370**(6520): p. 16.

8. Drouin AC, T.M., Liu SY, Smither AR, Flaherty SM, Zeller M, Geba GP, Reynaud P, Rothwell WB, Luk AP, Tian D, Boisen ML, Branco LM, Andersen KG, Robinson JE, Garry RF, Fusco DN., *Successful Clearance of 300 Day SARS-CoV-2 Infection in a Subject with B-Cell Depletion Associated Prolonged (B-DEAP) COVID by REGEN-COV Anti-Spike Monoclonal Antibody Cocktail.* Viruses, 2021. **13**(7).
9. Shi R, S.C., Duan X, Chen Z, Liu P, Song J, Song T, Bi X, Han C, Wu L, Gao G, Hu X, Zhang Y, Tong Z, Huang W, Liu WJ, Wu G, Zhang B, Wang L, Qi J, Feng H, Wang FS, Wang Q, Gao GF, Yuan Z, Yan J, *A human neutralizing antibody targets the receptor-binding site of SARS-CoV-2.* Nature, 2020. **584**(7819): p. 5.
10. Zhou T, W.L., Misasi J, Pegu A, Zhang Y, Harris DR, Olia AS, Talana CA, Yang ES, Chen M, Choe M, Shi W, Teng IT, Creanga A, Jenkins C, Leung K, Liu T, Stancofski ED, Stephens T, Zhang B, Tsybovsky Y, Graham BS, Mascola JR, Sullivan NJ, Kwong PD, *Structural basis for potent antibody neutralization of SARS-CoV-2 variants including B.1.1.529.* Science, 2022.
11. Gottlieb RL, N.A., Chen P, Boscia J, Heller B, Morris J, Huhn G, Cardona J, Mocherla B, Stosor V, Shawa I, Kumar P, Adams AC, Van Naarden J, Custer KL, Durante M, Oakley G, Schade AE, Holzer TR, Ebert PJ, Higgs RE, Kallewaard NL, Sabo J, Patel DR, Klekotka P, Shen L, Skovronsky DM, *Effect of Bamlanivimab as Monotherapy or in Combination With Etesevimab on Viral Load in Patients With Mild to Moderate COVID-19: A Randomized Clinical Trial.* JAMA, 2021. **325**(7): p. 13.
12. Jones BE, B.-A.P., Corbett KS, Westendorf K, Davies J, Cujec TP, Wiethoff CM, Blackbourne JL, Heinz BA, Foster D, Higgs RE, Balasubramaniam D, Wang L, Zhang Y, Yang ES, Bidshahri R, Kraft L, Hwang Y, Žentelis S, Jepson KR, Goya R, Smith MA, Collins DW, Hinshaw SJ, Tycho SA, Pellacani D, Xiang P, Muthuraman K, Sobhanifar S, Piper MH, Triana FJ, Hendle J, Pustilnik A, Adams AC, Berens SJ, Baric RS, Martinez DR, Cross RW, Geisbert TW, Borisevich V, Abiona O, Belli HM, de Vries M, Mohamed A, Dittmann M, Samanovic MI, Mulligan MJ, Goldsmith JA, Hsieh CL, Johnson NV, Wrapp D, McLellan JS, Barnhart BC, Graham BS, Mascola JR, Hansen CL, Falconer E., The neutralizing antibody, LY-CoV555, protects against SARS-CoV-2 infection in nonhuman primates. Sci Transl Med, 2021. 13(593).
13. Westendorf K, Ž.S., Wang L, Foster D, Vaillancourt P, Wiggin M, Lovett E, van der Lee R, Hendle J, Pustilnik A, Sauder JM, Kraft L, Hwang Y, Siegel RW, Chen J, Heinz BA, Higgs RE, Kallewaard NL, Jepson K, Goya R, Smith MA, Collins DW, Pellacani D, Xiang P, de Puyraimond V, Ricicova M, Devorkin L, Pritchard C, O'Neill A, Dalal K, Panwar P, Dhupar H, Garces FA, Cohen CA, Dye JM, Huie KE, Badger CV, Kobasa D, Audet J, Freitas JJ, Hassanali S, Hughes I, Munoz L, Palma HC, Ramamurthy B, Cross RW, Geisbert TW, Menachery V, Lokugamage K, Borisevich V, Lanz I, Anderson L, Sipahimalani P, Corbett KS, Yang ES, Zhang Y, Shi W, Zhou T, Choe M, Misasi J, Kwong PD, Sullivan NJ, Graham BS, Fernandez TL, Hansen CL, Falconer E, Mascola JR, Jones BE, Barnhart BC., LY-CoV1404 (bebtelovimab) potently neutralizes SARS-CoV-2 variants. Cell Rep, 2022. 39(7).
14. Pinto D, P.Y., Beltramello M, Walls AC, Tortorici MA, Bianchi S, Jaconi S, Culap K, Zatta F, De Marco A, Peter A, Guarino B, Spreafico R, Cameroni E, Case JB, Chen RE, Havenar-Daughton C, Snell G, Telenti A, Virgin HW, Lanzavecchia A, Diamond MS, Fink K, Veesler D, Corti D., *Cross-neutralization of SARS-CoV-2 by a human monoclonal SARS-CoV antibody.* Nature, 2020. **583**(7815): p. 6.
15. Rappazzo CG, T.L., Kaku CI, Wrapp D, Sakharkar M, Huang D, Deveau LM, Yockachonis TJ, Herbert AS, Battles MB, O'Brien CM, Brown ME, Geoghegan JC, Belk J, Peng L, Yang L, Hou Y, Scobey TD, Burton DR, Nemazee D, Dye JM, Voss JE, Gunn BM, McLellan JS, Baric RS, Gralinski

LE, Walker LM., *Broad and potent activity against SARS-like viruses by an engineered human monoclonal antibody.* Science, 2021. **371**(6531): p. 7.

16. Liu L, I.S., Guo Y, Chan JF, Wang M, Liu L, Luo Y, Chu H, Huang Y, Nair MS, Yu J, Chik KK, Yuen TT, Yoon C, To KK, Chen H, Yin MT, Sobieszczyk ME, Huang Y, Wang HH, Sheng Z, Yuen KY, Ho DD., *Striking antibody evasion manifested by the Omicron variant of SARS-CoV-2.* Nature, 2022. **602**(7898): p. 6.
17. Martinez DR, S.A., Gobeil S, Li D, De la Cruz G, Parks R, Lu X, Barr M, Stalls V, Janowska K, Beaudoin E, Manne K, Mansouri K, Edwards RJ, Cronin K, Yount B, Anasti K, Montgomery SA, Tang J, Golding H, Shen S, Zhou T, Kwong PD, Graham BS, Mascola JR, Montefiori DC, Alam SM, Sempowski G, Sempowski GD, Khurana S, Wiehe K, Saunders KO, Acharya P, Haynes BF, Baric RS., *A broadly cross-reactive antibody neutralizes and protects against sarbecovirus challenge in mice.* Sci Transl Med, 2022. **14**(629).
18. Pinto D, S.M., Czudnochowski N, Low JS, Tortorici MA, Housley MP, Noack J, Walls AC, Bowen JE, Guarino B, Rosen LE, di Iulio J, Jerak J, Kaiser H, Islam S, Jaconi S, Sprugasci N, Culap K, Abdelnabi R, Foo C, Coelmont L, Bartha I, Bianchi S, Silacci-Fregni C, Bassi J, Marzi R, Vetti E, Cassotta A, Ceschi A, Ferrari P, Cippà PE, Giannini O, Ceruti S, Garzoni C, Riva A, Benigni F, Cameroni E, Piccoli L, Pizzuto MS, Smithey M, Hong D, Telenti A, Lempp FA, Neyts J, Havenar-Daughton C, Lanzavecchia A, Sallusto F, Snell G, Virgin HW, Beltramello M, Corti D, Veesler D., *Broad betacoronavirus neutralization by a stem helix–specific human antibody.* Science, 2021. **373**(6559): p. 8.
19. Shi W, W.L., Zhou T, Sastry M, Yang ES, Zhang Y, Chen M, Chen X, Choe M, Creanga A, Leung K, Olia AS, Pegu A, Rawi R, Schön A, Shen CH, Stancofski ED, Talana CA, Teng IT, Wang S, Corbett KS, Tsybovsky Y, Mascola JR, Kwong PD., *Vaccine-elicited murine antibody WS6 neutralizes diverse beta-coronaviruses by recognizing a helical stem supersite of vulnerability.* Structure, 2022.
20. Claireaux M, C.T., de Gast M, Han J, Guerra D, Kerster G, van Schaik BDC, Jongejan A, Schriek AI, Grobben M, Brouwer PJM, van der Straten K, Aldon Y, Capella-Pujol J, Snitselaar JL, Olijhoek W, Aartse A, Brinkkemper M, Bontjer I, Burger JA, Poniman M, Bijl TPL, Torres JL, Copps J, Martin IC, de Taeye SW, de Bree GJ, Ward AB, Sliepen K, van Kampen AHC, Moerland PD, Sanders RW, van Gils MJ., *A public antibody class recognizes an S2 epitope exposed on open conformations of SARS-CoV-2 spike.* Nat Commun, 2022. **13**(1).

List of Supplementary Figures

Supplementary Figure 1. Immunoblots of the fractions of SARS-CoV-2 S6P-HBsAgs after ultracentrifugation

Supplementary Figure 2. Antibody binding of SARS-CoV-2 S6P-HBsAg nanoparticles

Supplementary Figure 3. Antibody binding of SARS-CoV-2 S2P and HBsAg

Supplementary Figure 4. BLI binding profiles of SARS-CoV-2 S6P-HBsAg nanoparticles

Supplementary Figure 5. IgG titers against SARS-CoV-2 S2P, RBD and NTD

Supplementary Figure 6. Neutralization ID80s against SARS-CoV-2 WA1 and variant pseudoviruses

Supplementary Figure 7. IgG titers against SARS-CoV-2 variant S2Ps

Supplementary Figure 8. Neutralization potency at weeks 6 and 14 elicited by SARS-CoV-2 S6P-HBsAgs

Supplementary Figure 9. Durability of anti-spike responses elicited by SARS-CoV-2 S6P-HBsAgs

Supplementary Figure 10. Durability of anti-HBsAg responses elicited by SARS-CoV-2 S6P-HBsAgs

Supplementary Figure 11. Durability of neutralizing antibody responses elicited by SARS-CoV-2 S6P-HBsAgs

Supplementary Figure 12. Anti-HBsAg endpoint titers in mice preimmunized with Recombivax HB

Supplementary Figure 13. IgG titers against SARS-CoV-2 WA1 and BA.1 S2Ps in mice preimmunized with Recombivax HB

Supplementary Figure 14. Neutralization potency in mice preimmunized with Recombivax HB

Supplementary Figure 15. Comparison of neutralization potency in mice with and without RecombiVax HB pre-vaccination

Supplementary Figure 1. Immunoblots of the fractions of SARS-CoV-2 S6P-HBsAgs after ultracentrifugation

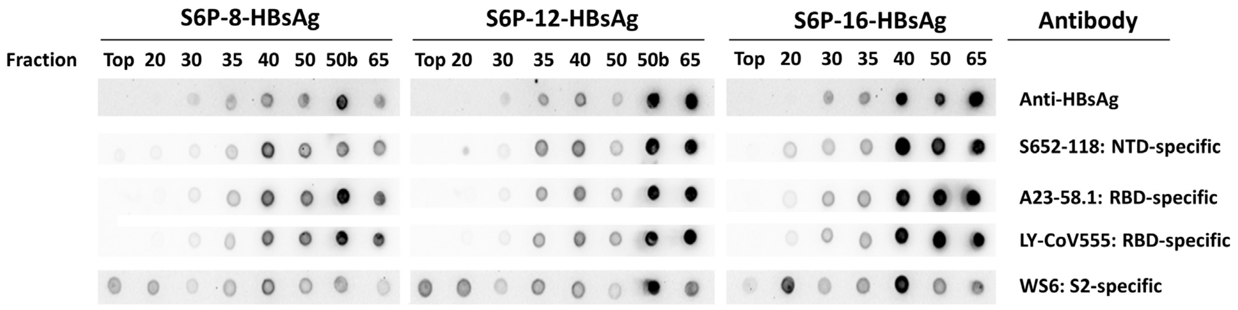

Supplementary Figure 1. Immunoblots of the fractions of SARS-CoV-2 S6P-HBsAgs after ultracentrifugation. Fractions of S6P-8-HBsAg, S6P-12-HBsAg and S6P-16-HBsAg after ultracentrifugation through a sucrose gradient were shown from left to right. Two microliters of each sucrose fraction were spotted onto a nitrocellulose membrane and then blocked with 5% skim milk in phosphate buffered saline-tweeen (PBST) at room temperature for 1 hour. The membrane was washed with 15 ml PBST solution thrice. Following the washes, the membrane was incubated with 1 μg/ml mAb specific to the NTD, RBD or S2 domain of SARS-CoV-2 spike, in 10 ml of 5% skim milk in PBST at room temperature for 30 min. The membrane was then washed thrice. The horse radish peroxidase (HRP)-conjugated anti-human or anti-mouse secondary antibody (1/1000) was incubated with the membrane for 30 min at room temperature. The GE ECL kit was used to develop the membrane. The fractions were shown above the blots. “Top” refers to the layer laid on the top of the 20% sucrose cushion. Numbers denote the percentage of the sucrose solution in a buffer containing 20 mM MES, pH 6.0, 150 mM NaCl. “50b” stands for the band in 50% sucrose after ultracentrifugation. The mAbs S652-118, A23-58.1, LY-CoV555 and WS6, which are specific to the NTD, RBD and S2 domain of SARS-CoV-2 spike, respectively, were used and indicated on the right.

Supplementary Figure 2. Antibody binding of SARS-CoV-2 S6P-HBsAg nanoparticles

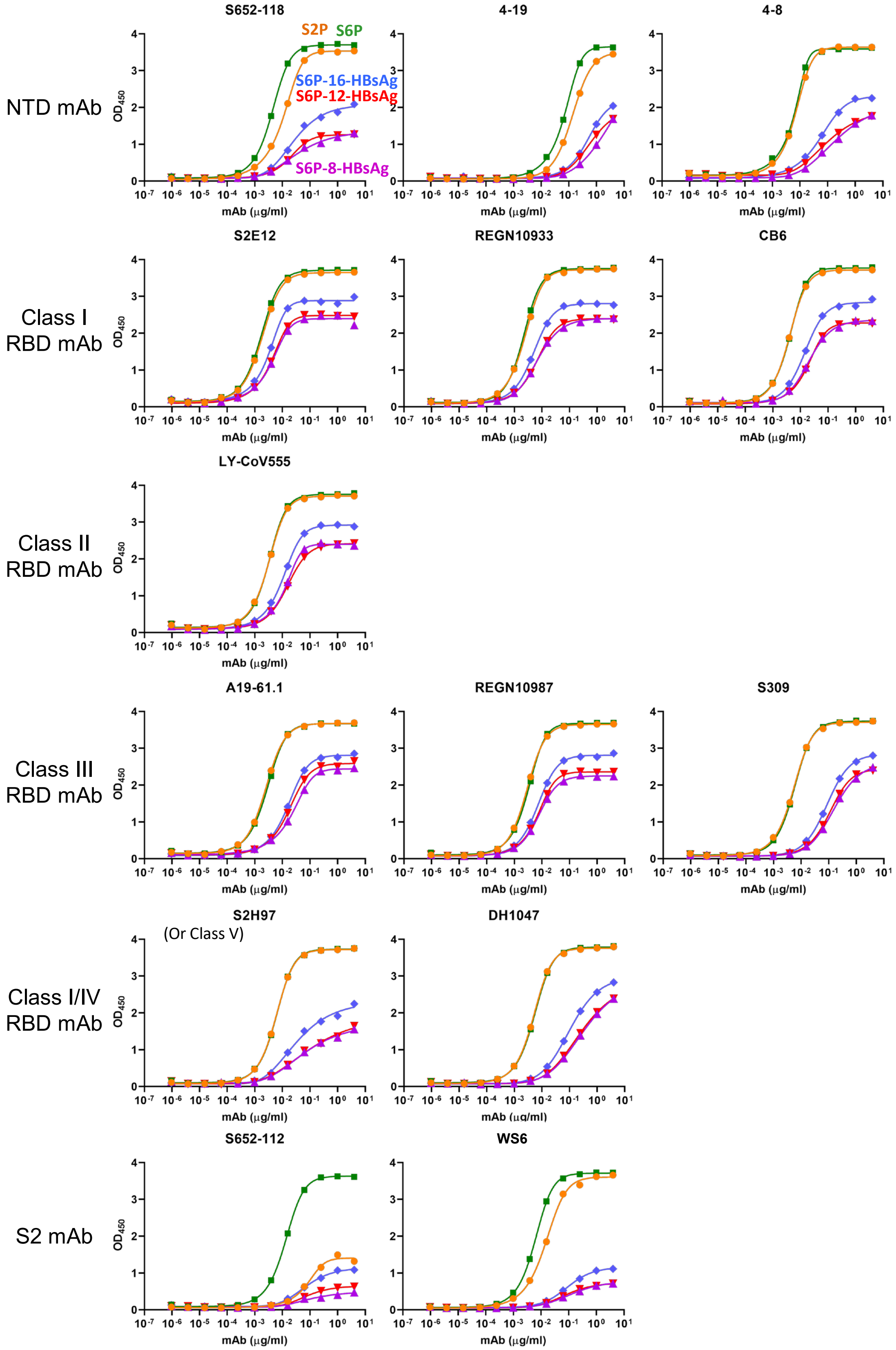

Supplementary Figure 2. Antibody binding of SARS-CoV-2 S6P-HBsAg nanoparticles. ELISA binding to mAbs specific to different domains of SARS-CoV-2 spike was performed using plates coated with 1 µg/ml SARS-CoV-2 S2P (orange), S6P (green), S6P-8-HBsAg (purple), S6P-12-HBsAg (red) or S6P-16-HBsAg (blue). Antibodies were tested from 0.95 pg/ml to 4 µg/ml. Goat anti-human or anti-mouse IgG conjugated with horseradish peroxidase (1/2000) was used as the secondary antibody. Incubation with primary and secondary antibodies were done at room temperature for 1 h. Plates were developed using TMB at room temperature for 10 min, stopped with 1N H_2SO_4. Optical density at 450 nm were measured and plotted against mAb concentrations shown in a logarithmic scale. The classification of the mAbs was indicated on the left for plots in each row.

Supplementary Figure 3. Antibody binding of SARS-CoV-2 S2P and HBsAg

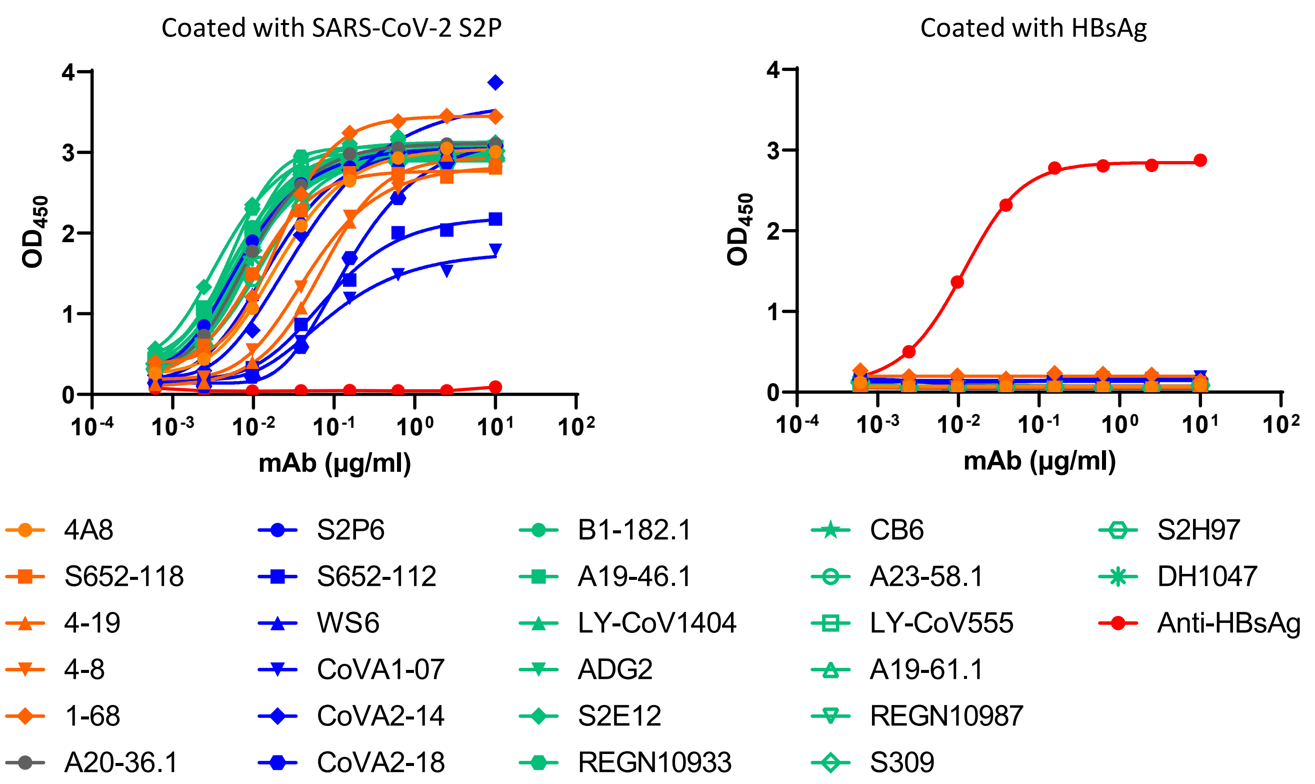

Supplementary Figure 3. Antibody binding of SARS-CoV-2 S2P and HBsAg. ELISA binding of SARS-CoV-2 mAbs was performed using plates coated with 1 µg/ml SARS-CoV-2 S2P (left) or HBsAg (right). Antibodies were tested from 0.61 ng/ml to10 µg/ml. The plots were shown in orange, grey, blue, green and red for the mAbs specific to the NTD, SD1, S2 and RBD domains of SARS-CoV-2 spike and anti-HBsAg antibody, respectively. The legend for each mAb was shown below the plots. Goat anti-human or anti-mouse IgG conjugated with horseradish peroxidase (1/2000) was used as the secondary antibody. Incubation with primary and secondary antibodies were done at room temperature for 1 h. Plates were developed using TMB at room temperature for 5 min, stopped with 1N H_2SO_4. Optical density at 450 nm were measured and plotted against mAb concentrations shown in a logarithmic scale.

Supplementary Figure 4. BLI binding profiles of SARS-CoV-2 S6P-HBsAg nanoparticles

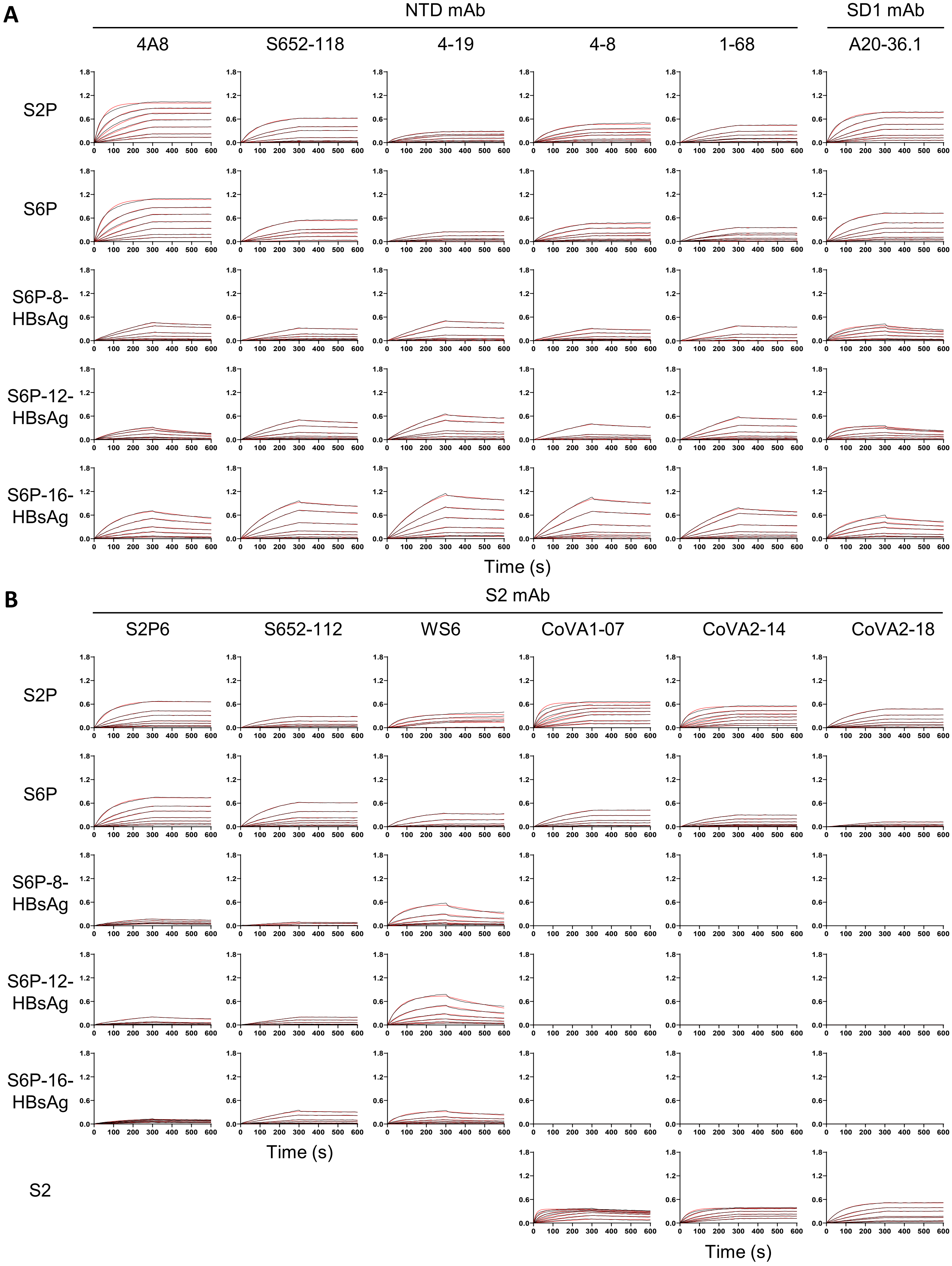

C

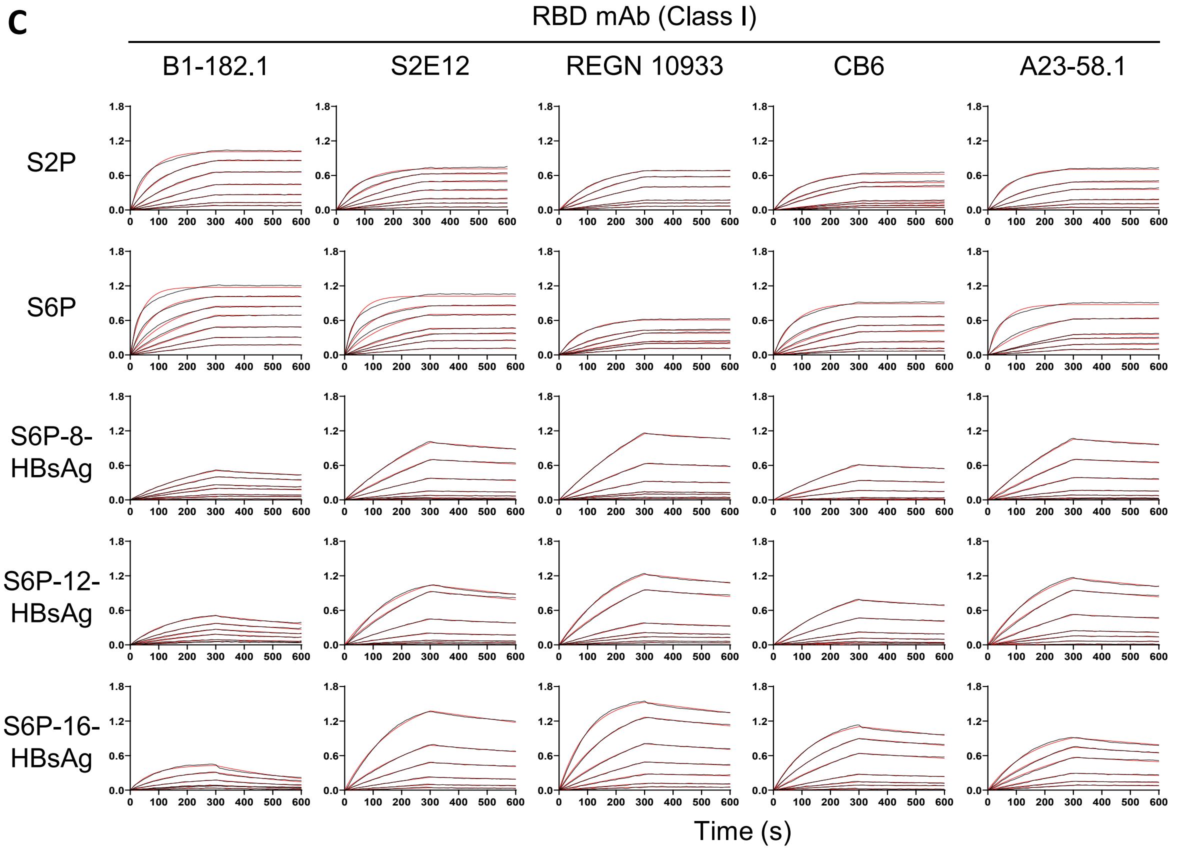
RBD mAb (Class I)
B1-182.1
S2E12
REGN 10933
CB6
A23-58.1
S2P
S6P
S6P-8-HBsAg
S6P-12-HBsAg
S6P-16-HBsAg
Time (s)

D

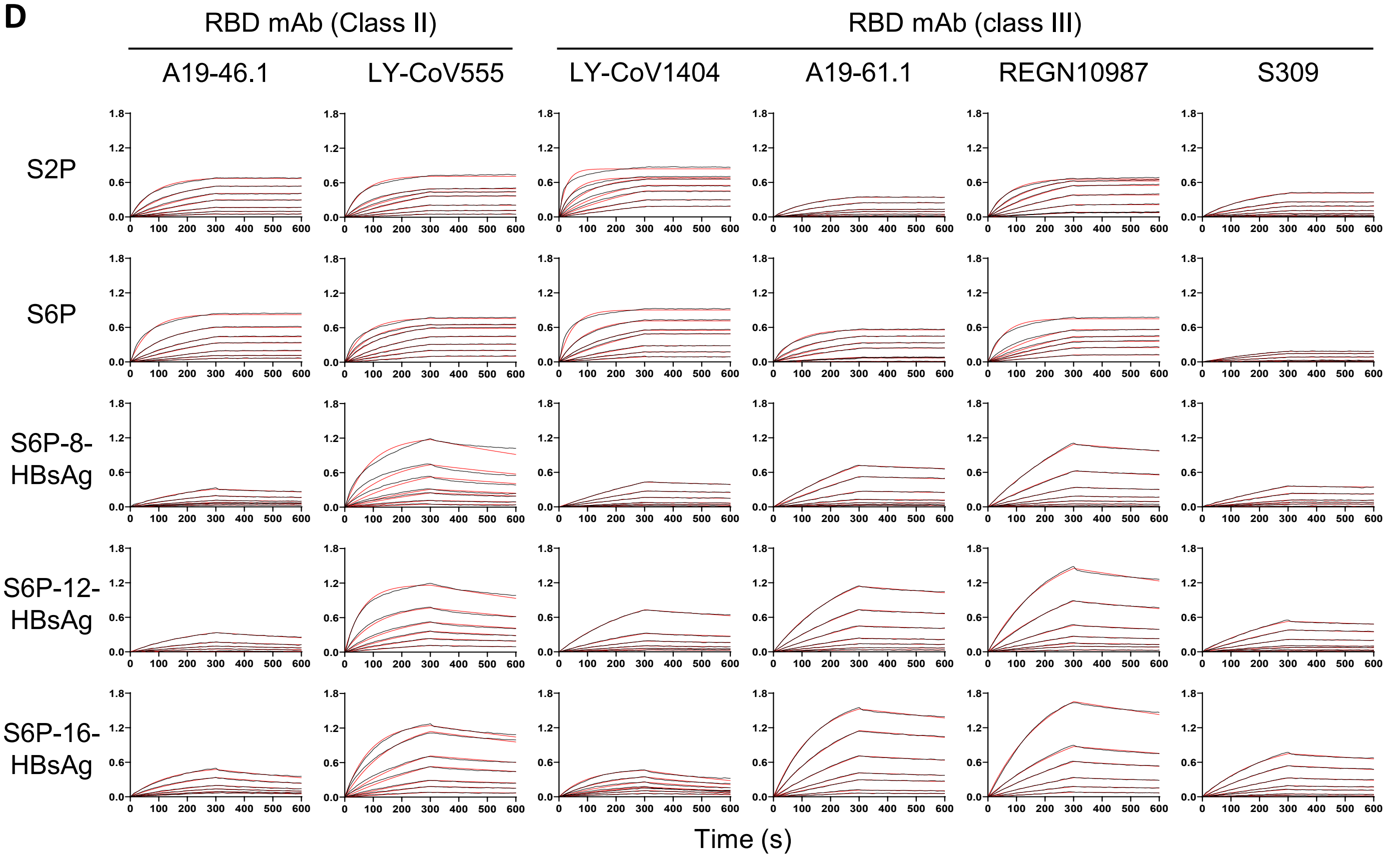
RBD mAb (Class II)
RBD mAb (class III)
A19-46.1
LY-CoV555
LY-CoV1404
A19-61.1
REGN10987
S309
S2P
S6P
S6P-8-HBsAg
S6P-12-HBsAg
S6P-16-HBsAg
Time (s)

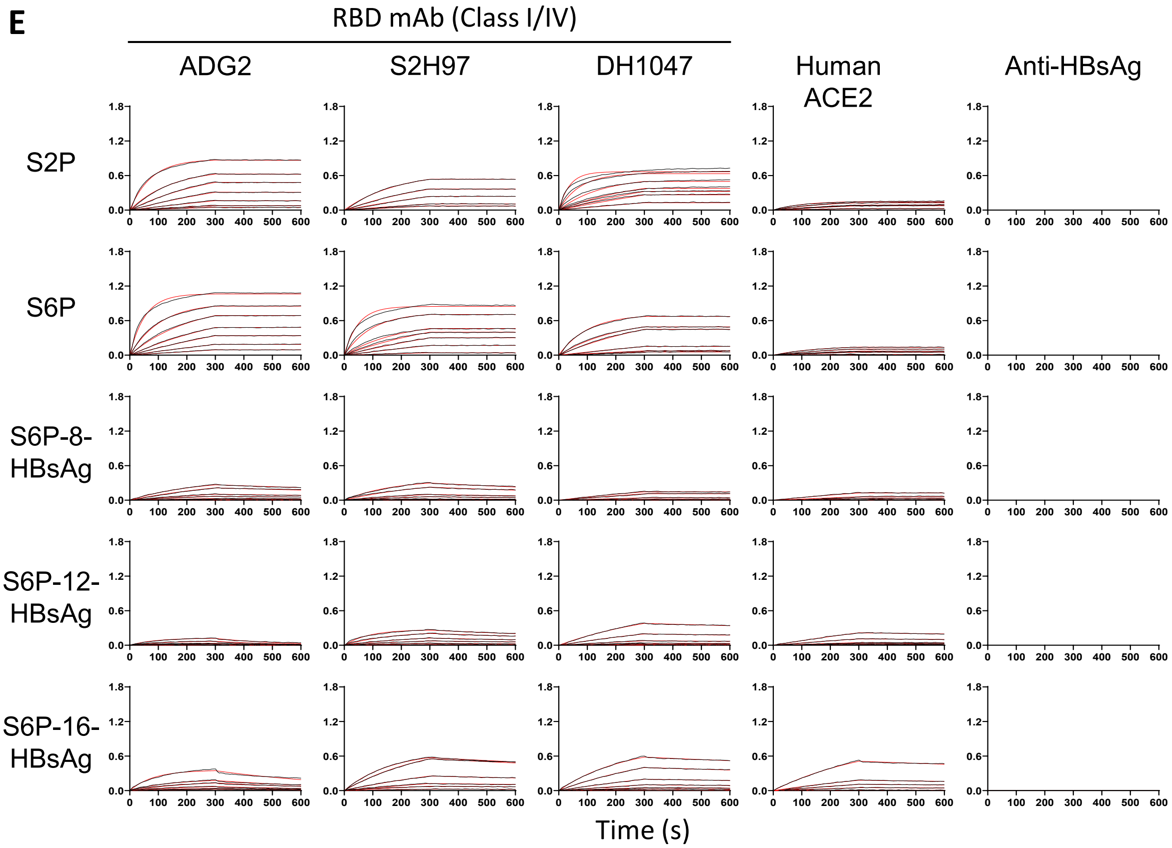

Supplementary Figure 4. BLI binding profiles of SARS-CoV-2 S6P-HBsAg nanoparticles. The binding of SARS-CoV-2 S2P, S6P, S6P-8-HBsAg, S6P-12-HBsAg and S6P-16-HBsAg to a panel of SARS-CoV-2 mAbs or human ACE2 was measured by an Octet HTX instrument. The binding buffer contains 1x HBS-EP+ buffer (GE) and 5% sucrose. The mAbs specific to the NTD and SD1 (A), S2 (B), and RBD domains (class I (C), classes II and III (D), class I/IV (E) or human ACE2 (E) with a Fc tag were captured by AHC or AMC sensors to yield a binding signal of 1~1.3 nm. The SARS-CoV-2 stabilized spike free or displayed on an HBsAg core varied from 0 to 800 nM. The molar concentration of the S6P-HBsAg was determined based on spike (but not based on a nanoparticle). The association and dissociation were done for 300 s, respectively. The binding curves were shown in black, and the global curve fitting with a 1:1 model using Data Analysis Software v9.0 was shown in red.

Supplementary Figure 5. IgG titers against SARS-CoV-2 S2P, RBD and NTD

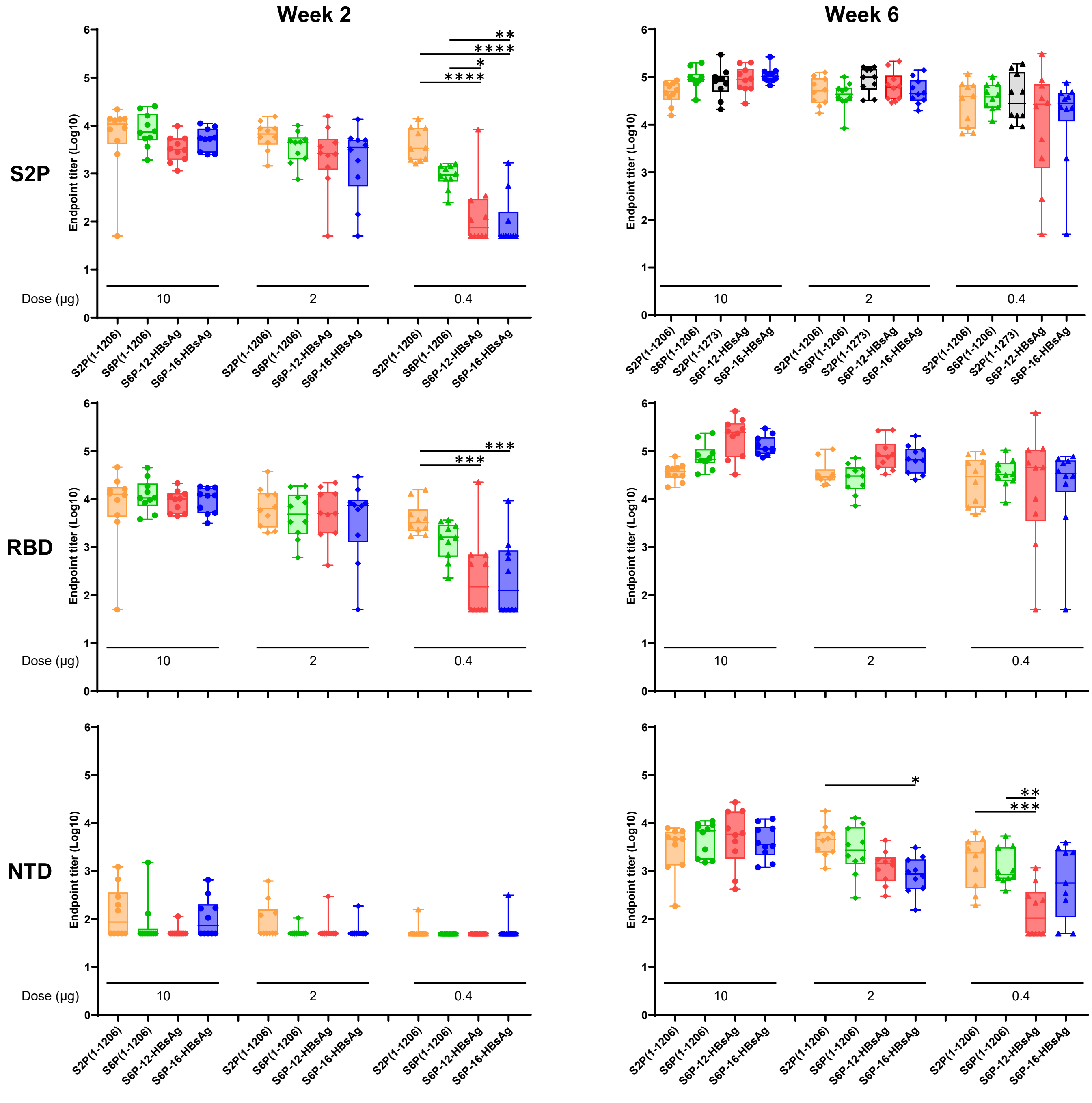

Supplementary Figure 5. IgG titers against SARS-CoV-2 S2P, RBD and NTD. The sera at 2 weeks post the 1st and 2nd immunizations from mice originally naïve to HBsAg were tested. The coating WA1 protein or domains were listed on the left. Endpoint IgG titers were plotted in a logarithmic scale in the box and whiskers format. The data points in the median quartile of each group were boxed. The error bars represent 95% confidence interval. The data points were shown in beige, green, black, red and blue for S2P(1-1206), S6P(1-1206), S2P(1-1273), S6P-12-HBsAg and S6P-16-HBsAg, respectively. The data for 10, 2 and 0.4 µg doses were plotted as dots, diamonds and triangles, respectively. The doses and immunogens were indicated. Statistical analyses were performed using two-way ANOVA test. * $p < 0.05$; ** $p < 0.01$; *** $p < 0.001$; **** $p < 0.0001$.

Supplementary Figure 6. Neutralization ID80s against SARS-CoV-2 WA1 and variant pseudoviruses

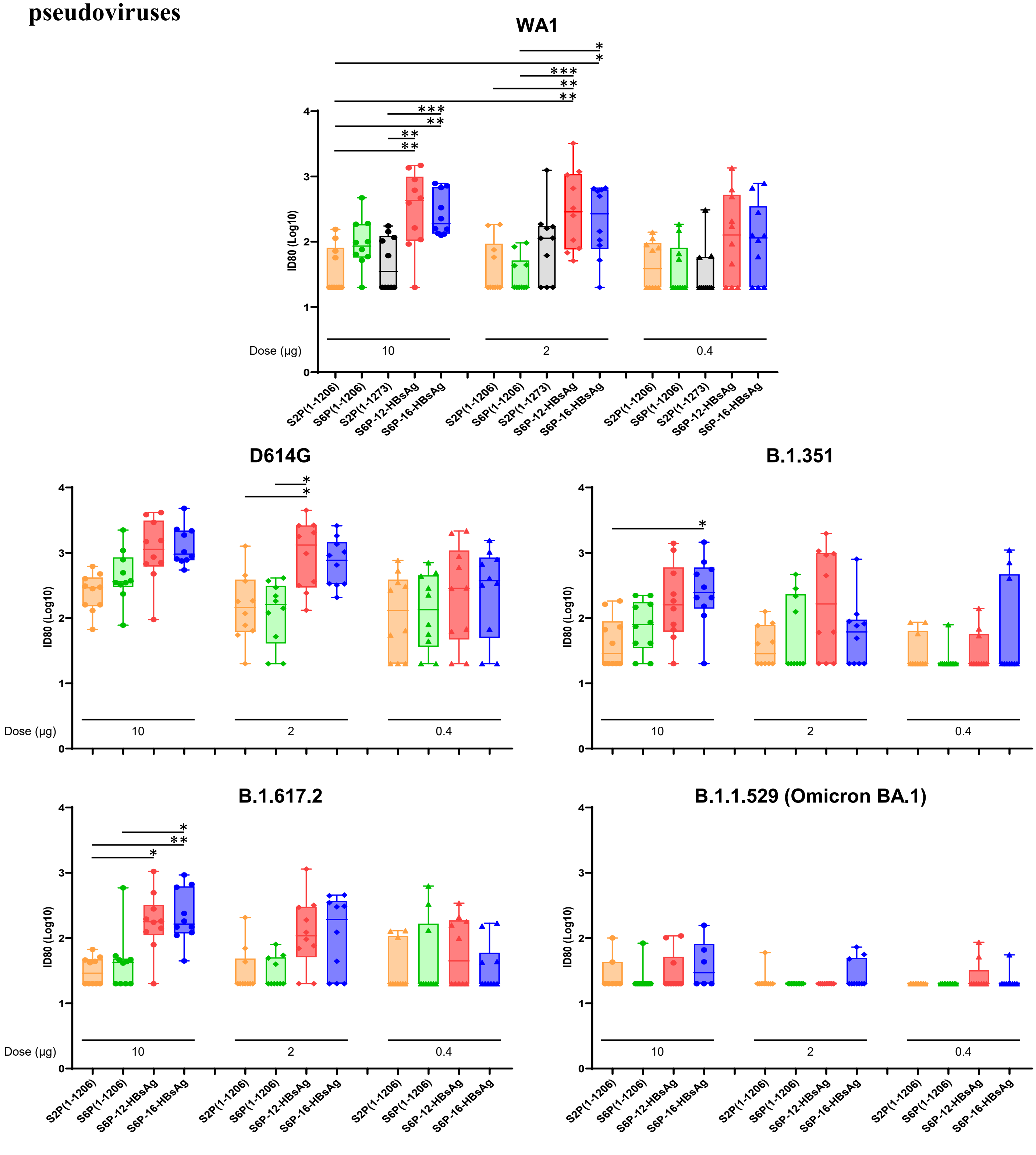

Supplementary Figure 6. Neutralization ID80s against SARS-CoV-2 WA1 and variant pseudoviruses. The sera at 2 weeks post the second immunization were evaluated against SARS-CoV-2 WA1, D614G, B.1.351, B.1.617.2 and B.1.1.529 pseudoviruses. The ID80s were plotted in a logarithmic scale in the box and whiskers format. The data points in the median quartile of each group were boxed. The error bars represent 95% confidence interval. The doses and immunogens were indicated. The data were plotted as dots, diamonds or triangles for 10, 2, and 0.4 μg doses, respectively. The data points were shown in beige, green, black, red and blue for S2P(1-1206), S6P-(1-1206), S2P(1-1273), S6P-12-HBsAg and S6P-12-HBsAg, respectively. The statistical analyses were performed using the two-way ANOVA test. * $p < 0.05$; ** $p < 0.01$; *** $p < 0.001$.

Supplementary Figure 7. IgG titers against SARS-CoV-2 variant S2Ps

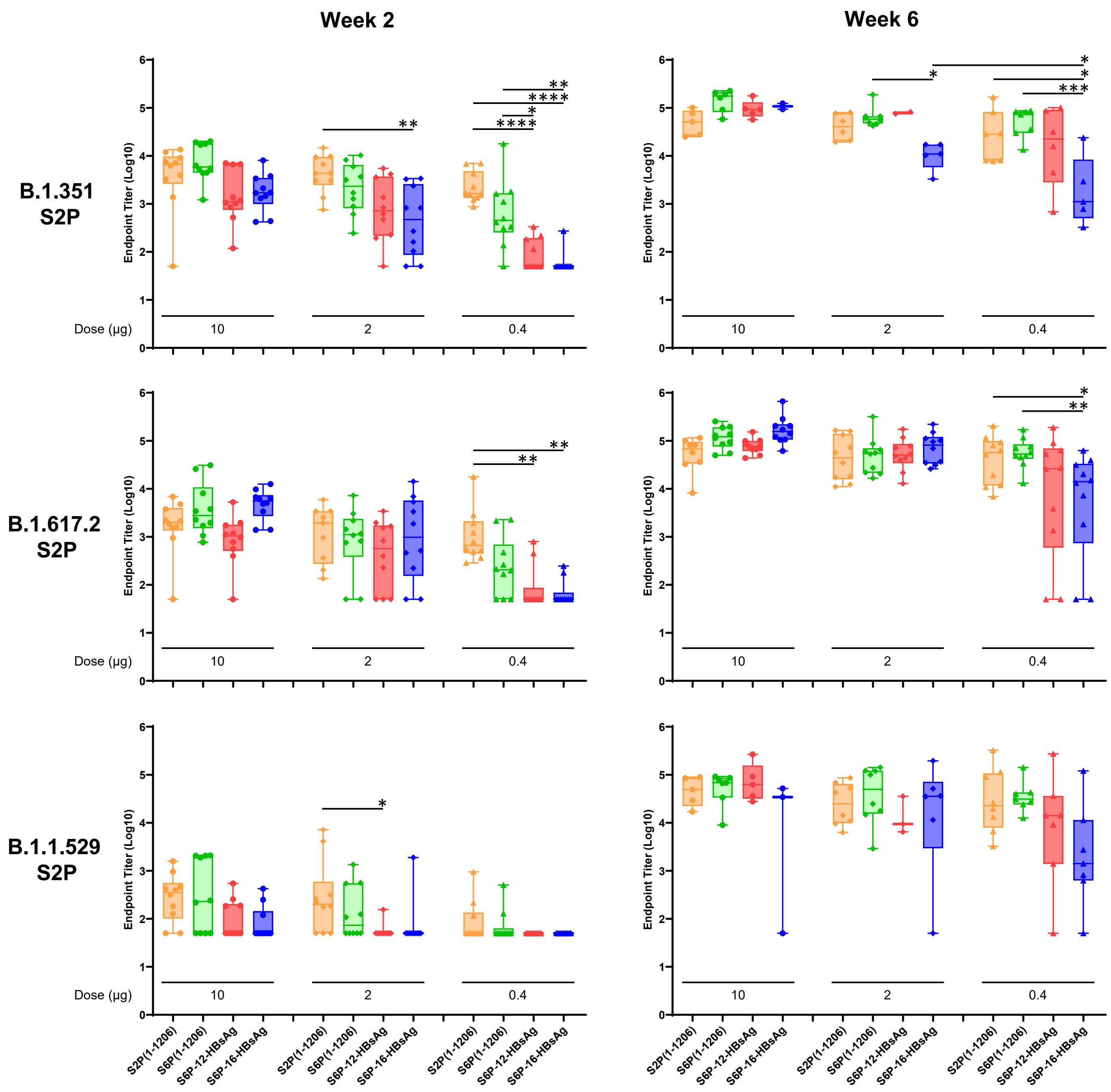

Supplementary Figure 7. IgG titers against SARS-CoV-2 variant S2Ps. Week 2 (left) and week 6 (right) sera from mice at 2 weeks post the 1st or 2nd DNA immunizations plus electroporation were tested. Endpoint IgG titers were plotted in a logarithmic scale in the box and whiskers format. The data points in the median quartile of each group were boxed. The error bars represent 95% confidence interval. The data for 10, 2 and 0.4 µg doses were shown as dots, diamonds and triangles, respectively. The data points were colored in beige, green, red and blue for S2P(1-1206), S6P(1-1206), S6P-12-HBsAg and S6P-16-HBsAg, respectively. The doses and immunogens were indicated below the plots. Statistical analyses were performed using two-way ANOVA test. * $p < 0.05$; ** $p < 0.01$; *** $p < 0.001$; **** $p < 0.0001$. Less data points were shown for some groups due to the unavailability of the sera for the missing ones. Data points less than 5 in a group were not included in the statistical analyses.

Supplementary Figure 8. Neutralization potency at weeks 6 and 14 elicited by SARS-CoV-2 S6P-HBsAgs

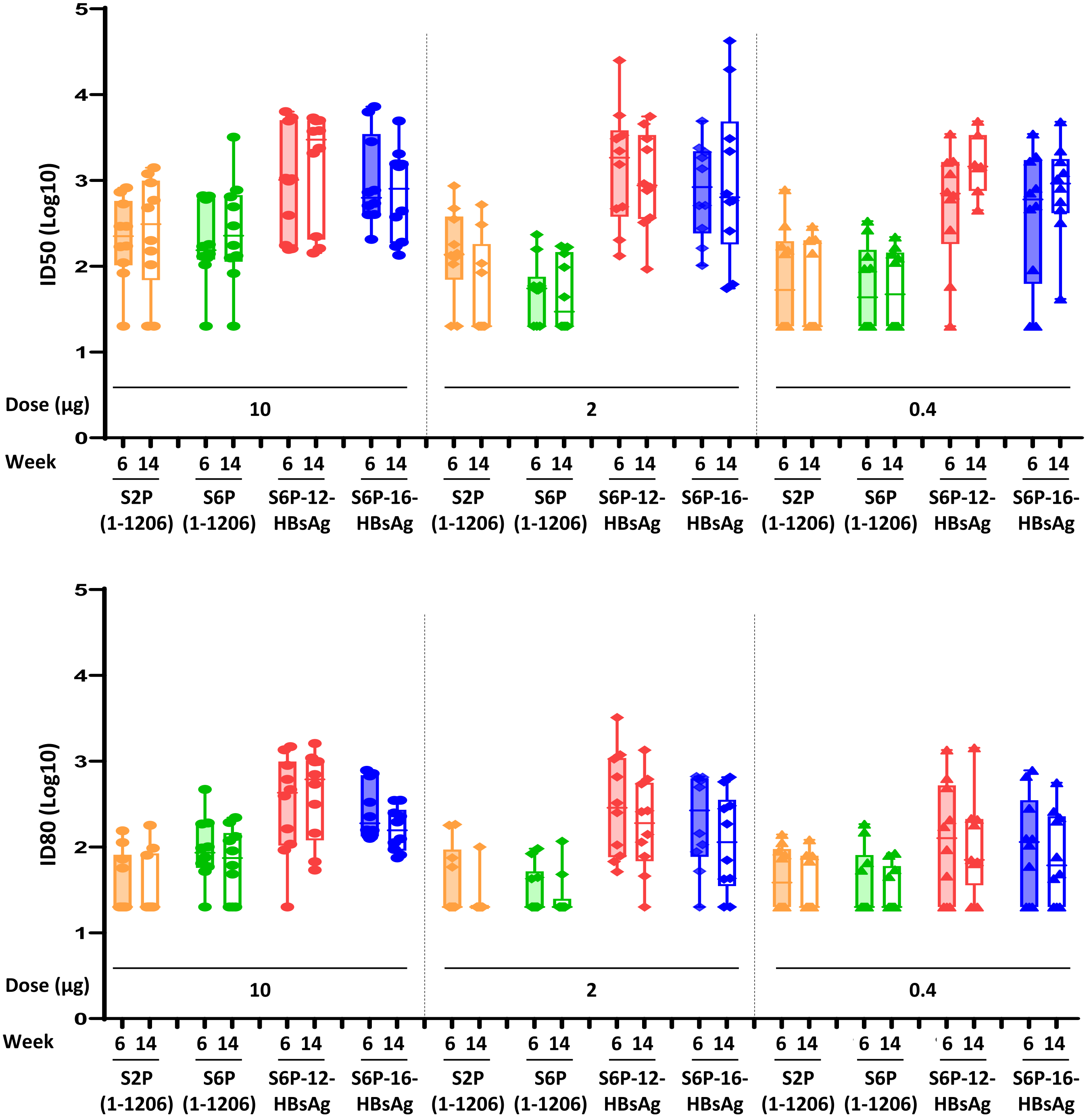

Supplementary Figure 8. Neutralization potency at weeks 6 and 14 elicited by SARS-CoV-2 S6P-HBsAgs. Week 6 and 14 sera after two DNA immunizations plus electroporation were tested. WA1 ID50 (top) and ID80s (bottom) were plotted in a logarithmic scale in the box and whiskers format. The data points in the median quartile in each group were boxed. The error bars indicate 95% confidence interval. The data for 10, 2 and 0.4 µg doses were plotted as dots, diamonds and triangles, respectively. The data points were shown in beige, green, red and blue for S2P(1-1206), S6P(1-1206), S6P-12-HBsAg and S6P-16-HBsAg, respectively.

Supplementary Figure 9. Durability of anti-spike responses elicited by SARS-CoV-2 S6P-HBsAgs

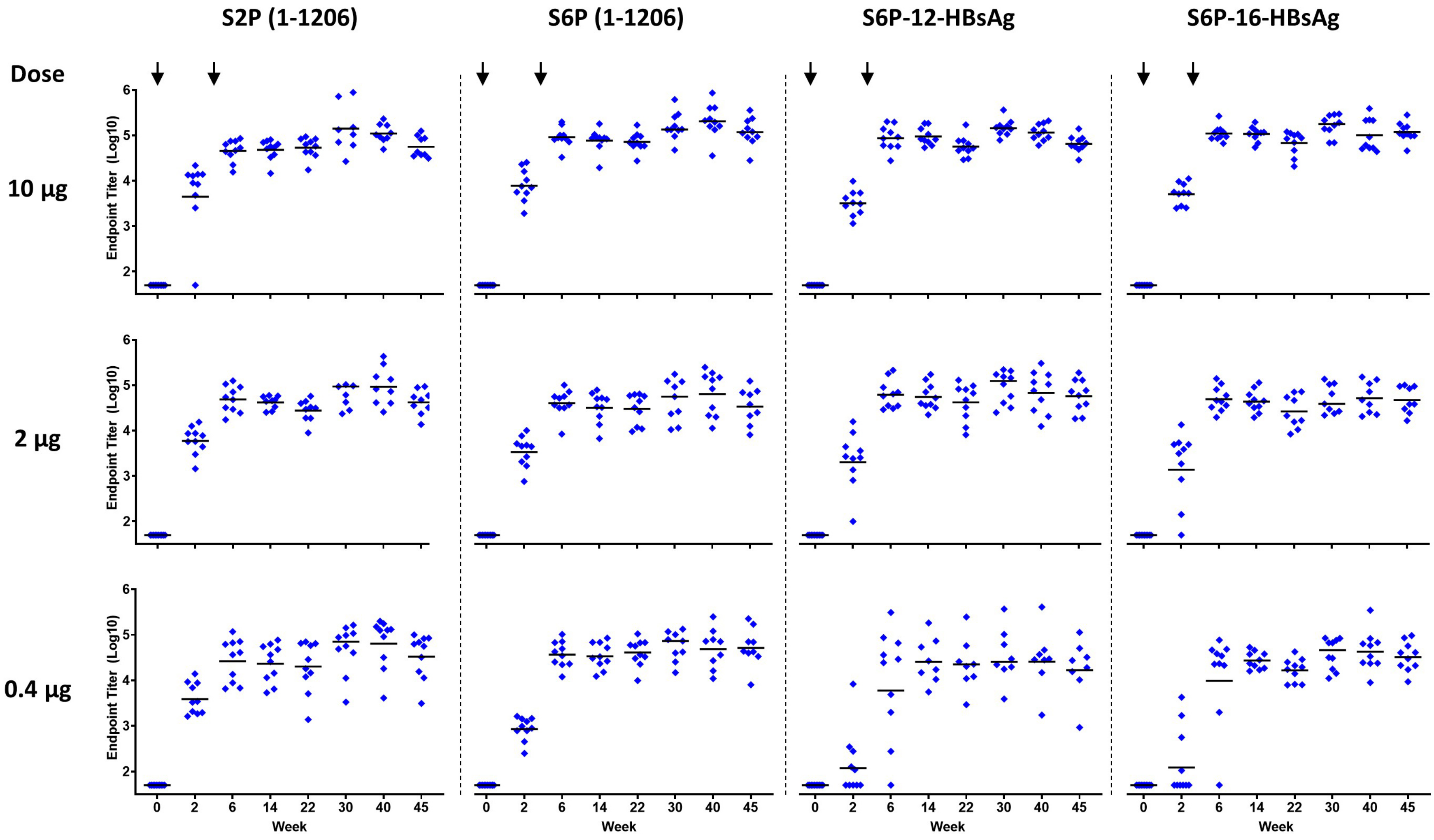

Supplementary Figure 9. Durability of anti-spike responses elicited by SARS-CoV-2 S6P-HBsAg. The endpoint IgG titers against WA1 S2P from week 0 to week 45 were shown from left to right for mice immunized twice with S2P(1-1206), S6P(1-1206), S6P-12-HBsAg or S6P-16-HBsAg. 10, 2 and 0.4 µg groups were shown from top to bottom. The endpoint IgG titer from each animal was shown as an individual diamond. The geometric mean titer of each group was shown as a black horizontal bar. Arrows indicate DNA immunizations at week 0 and 4 followed by electroporation.

Supplementary Figure 10. Durability of anti-HBsAg responses elicited by SARS-CoV-2 S6P-HBsAgs

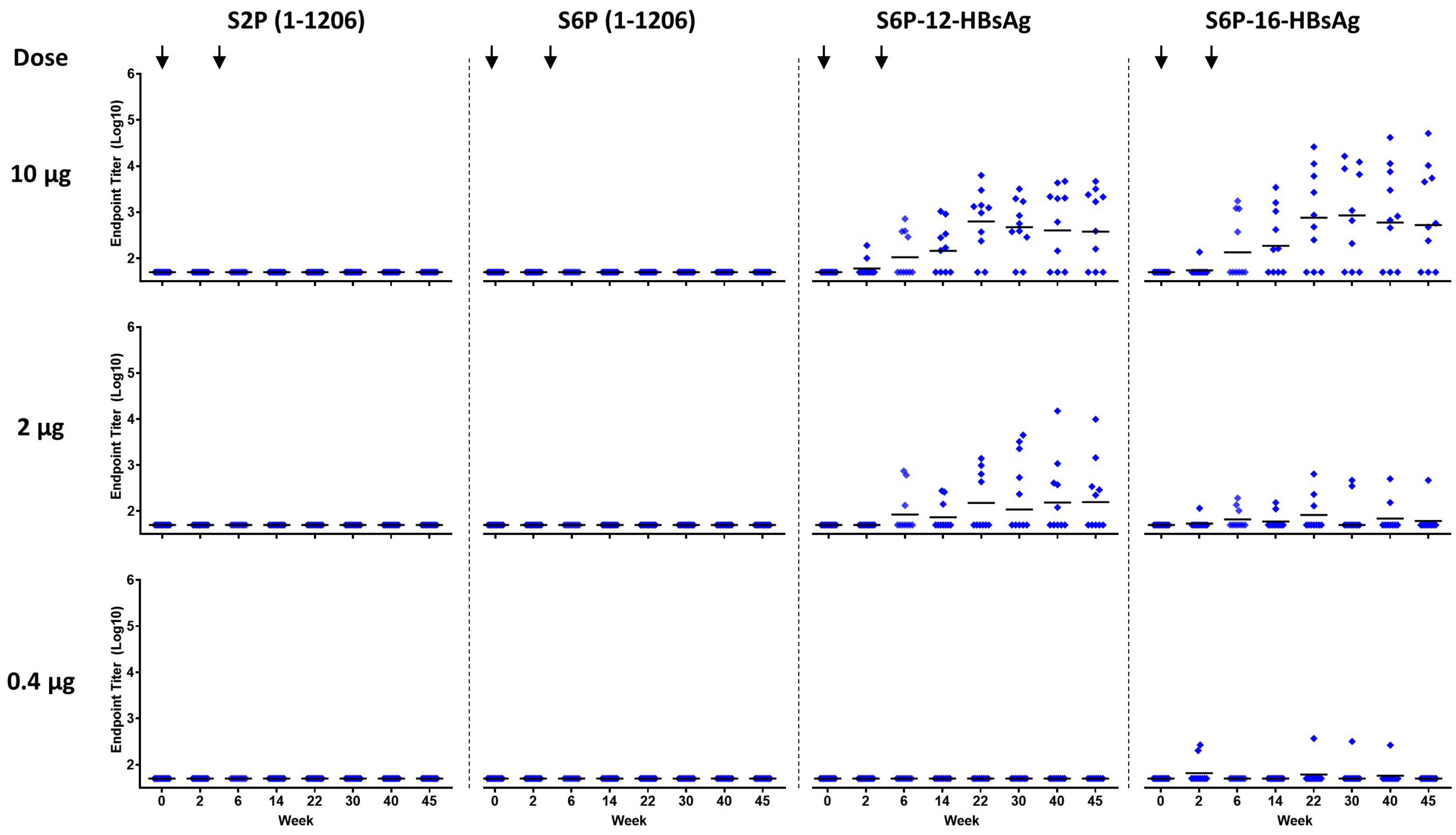

Supplementary Figure 10. Durability of anti-HBsAg responses elicited by SARS-CoV-2 S6P-HBsAg. The endpoint titers against HBsAg from week 0 to week 45 were shown from left to right for mice immunized twice with S2P(1-1206), S6P(1-1206), S6P-12-HBsAg or S6P-16-HBsAg. 10, 2 and 0.4 µg groups were shown from top to bottom. The endpoint IgG titer from each animal was shown as an individual diamond. The geometric mean titer of each group was shown as a black horizontal bar. Arrows indicate DNA immunizations at weeks 0 and 4 followed by electroporation.

Supplementary Figure 11. Durability of neutralizing antibody responses elicited by SARS-CoV-2 S6P-HBsAgs

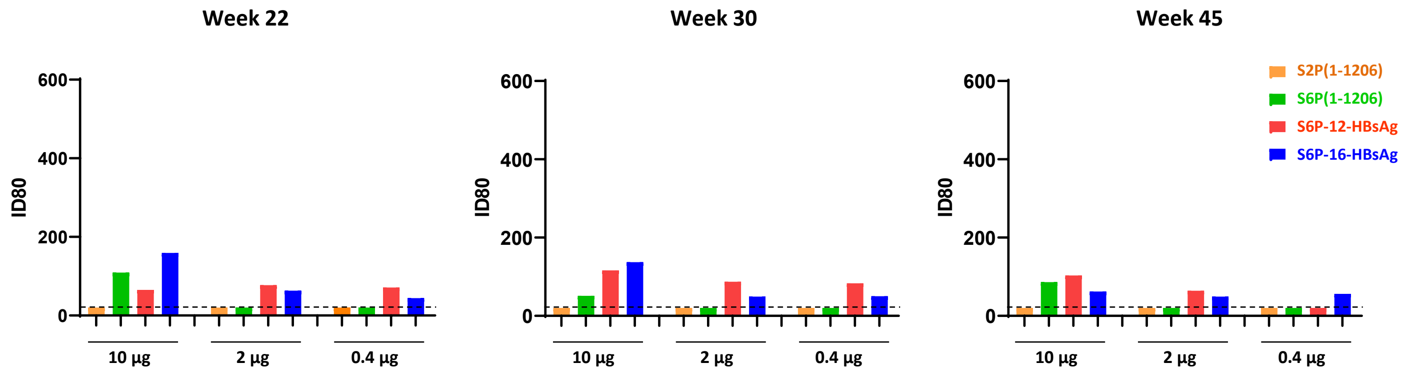

Supplementary Figure 11. Durability of neutralizing antibody responses elicited by SARS-CoV-2 S6P-HBsAgs. The sera from mice immunized with DNA encoding SARS-CoV-2 S2P(1-1206), S6P(1-1206), S6P-12-HBsAg or S6P-16-HBsAg were tested from week 22 to week 45. The ID80s against WA1 pseudovirus obtained from the pooled serum of each group were shown for the 10, 2 and 0.4 µg doses. The bars were colored in beige, green, red and blue for S2P(1-1206), S6P(1-1206), S6P-12-HBsAg and S6P-16-HBsAg, respectively. The graphs for weeks 22, 30 and 45 were shown in different panels. The dashed lines indicate detection limit.

Supplementary Figure 12. Anti-HBsAg endpoint titers in mice preimmunized with Recombivax HB

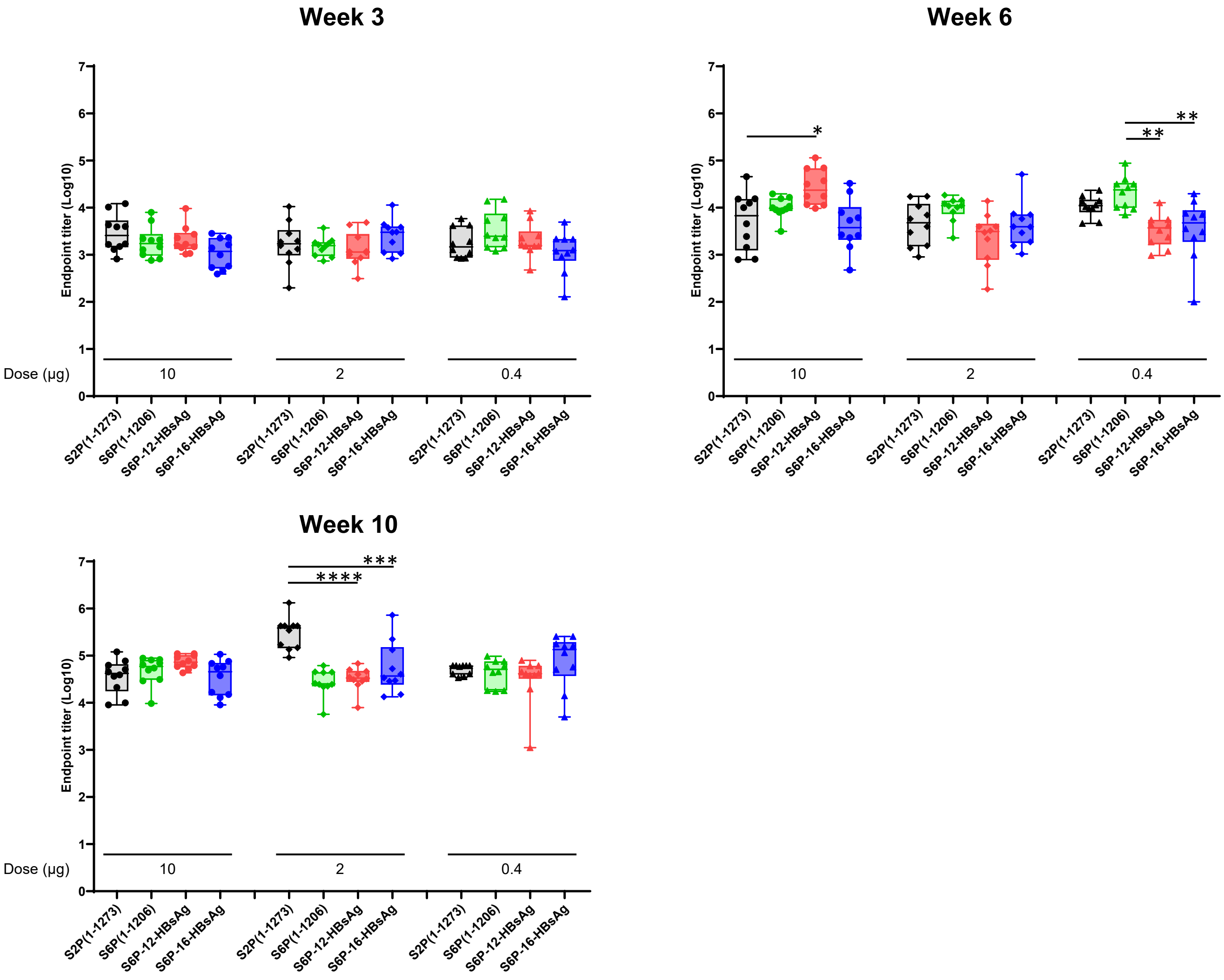

Supplementary Figure 12. Anti-HBsAg endpoint titers in mice preimmunized with Recombivax HB. Week 3, 6 and 10 sera from mice preimmunized with Recombivax HB followed by one or two DNA immunizations plus electroporation were tested. Endpoint IgG titers were plotted in a logarithmic scale in the box and whiskers format. The data points in the median quartile in each group were boxed. The error bars indicate 95% confidence interval. The data for 10, 2 and 0.4 µg doses were plotted as dots, diamonds and triangles, respectively. The data points were shown in black, green, red and blue for S2P(1-1273), S6P(1-1206), S6P-12-HBsAg and S6P-16-HBsAg, respectively. The doses and immunogens were indicated. Statistical analyses were performed using the two-way ANOVA test. * $p < 0.05$; ** $p < 0.01$; *** $p < 0.001$; **** $p < 0.0001$.

Supplementary Figure 13. IgG titers against SARS-CoV-2 WA1 and BA.1 S2Ps in mice preimmunized with Recombivax HB

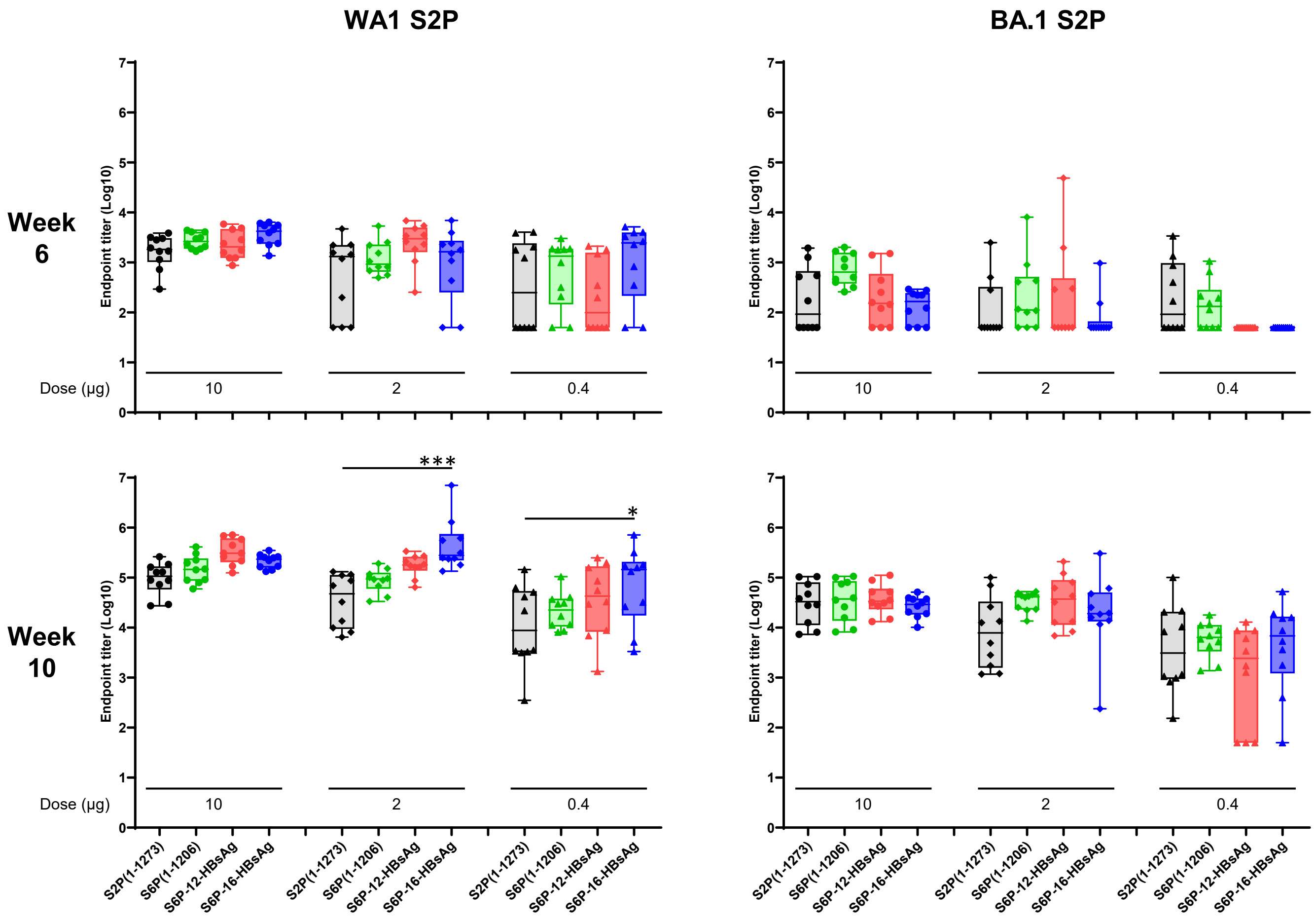

Supplementary Figure 13. IgG titers against SARS-CoV-2 WA1 and BA.1 S2Ps in mice preimmunized with Recombivax HB. Week 6 and 10 sera from mice preimmunized with Recombivax HB followed by one or two DNA immunizations plus electroporation were tested. WA1 and BA.1 S2Ps were used to coat plates and indicated on the top, respectively. Endpoint IgG titers were plotted in a logarithmic scale in the box and whiskers format. The data points in the median quartile of each group were boxed. Error bars represent 95% confidence interval. The data were shown in black, green, red and blue for S2P(1-1273), S6P(1-1206), S6P-12-HBsAg and S6P-16-HBsAg, respectively. The data for 10, 2 and 0.4 µg doses were shown as dots, diamonds and triangles, respectively. The doses and immunogens were indicated. Statistical analyses were performed using the two-way ANOVA test. * $p < 0.05$; ** $p < 0.01$; *** $p < 0.001$; **** $p < 0.0001$.

Supplementary Figure 14. Neutralization potency in mice preimmunized with Recombivax HB

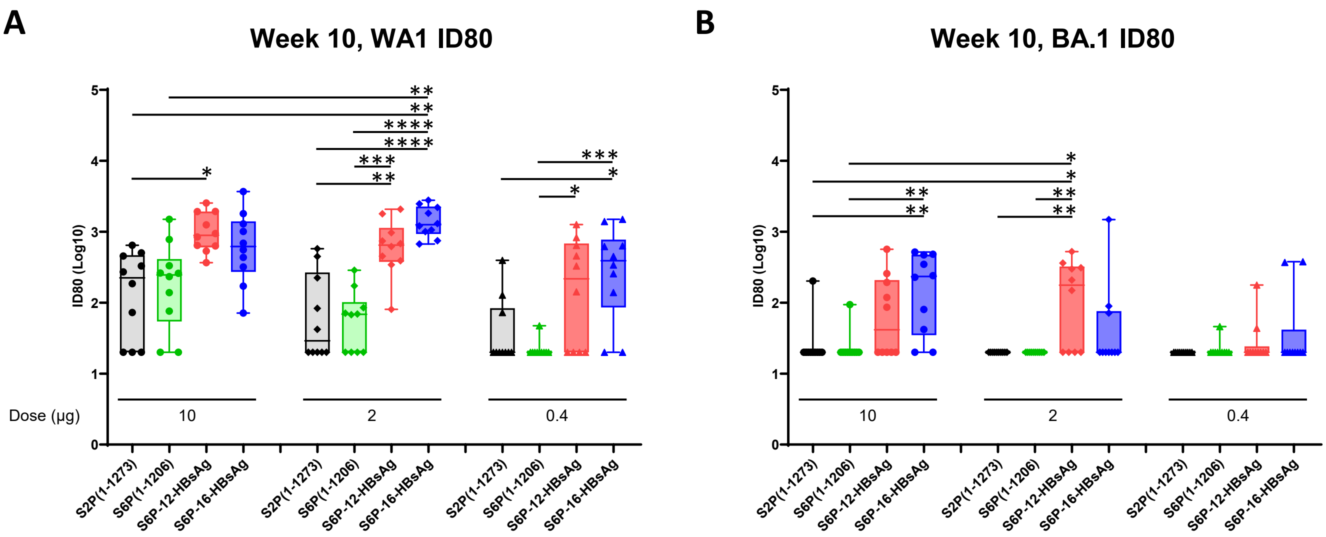

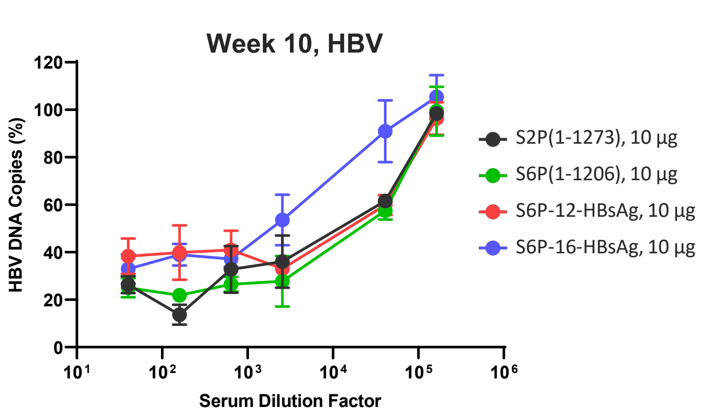

		HBV	
DNA	Dose (µg)	ID50	ID80
S2P(1-1273)	10	139434	54589
S6P(1-1206)	10	64711	196
S6P-12-HBsAg	10	43047	58
S6P-16-HBsAg	10	15751	84

Supplementary Figure 14. Neutralization potency in mice preimmunized with Recombivax HB. (A) ID80s against SARS-CoV-2 WA1 pseudovirus. (B) ID80s against BA.1 pseudovirus. The sera at 2 weeks post the second DNA immunization plus electroporation from mice preimmunized with Recombivax HB were evaluated. The ID80s were plotted in a logarithmic scale in the box and whiskers format. The data points in the median quartile in each group were boxed. Error bars represent 95% confidence interval. The doses and immunogens were indicated. The ID80s were plotted as dots, diamonds and triangles for 10, 2, and 0.4 µg doses, respectively. The data points were shown in black, green, red and blue for S2P(1-1273), S6P-(1-1206), S6P-12-HBsAg and S6P-12-HBsAg, respectively. The statistical analyses were performed using the two-way ANOVA test. * $p < 0.05$; ** $p < 0.01$; *** $p <0.001$; **** $p < 0.0001$. (C) Neutralization potency against HBV for sera at 2 weeks post the second DNA immunization plus electroporation from mice preimmunized with Recombivax HB. Normalized HBV DNA copies to viral control DNA copies in percentage were plotted against serum dilution factors. The plots in black, green, red and blue were shown on the left for 10 µg S2P(1-1273), S6P(1-1206), S6P-12-HBsAg and S6P-16-HBsAg, respectively. The data were obtained using pooled sera from each group (n = 10). The ID50 and ID80 titers were listed on the right.

Supplementary Figure 15. Comparison of neutralization potency in mice with and without Recombivax HB pre-vaccination

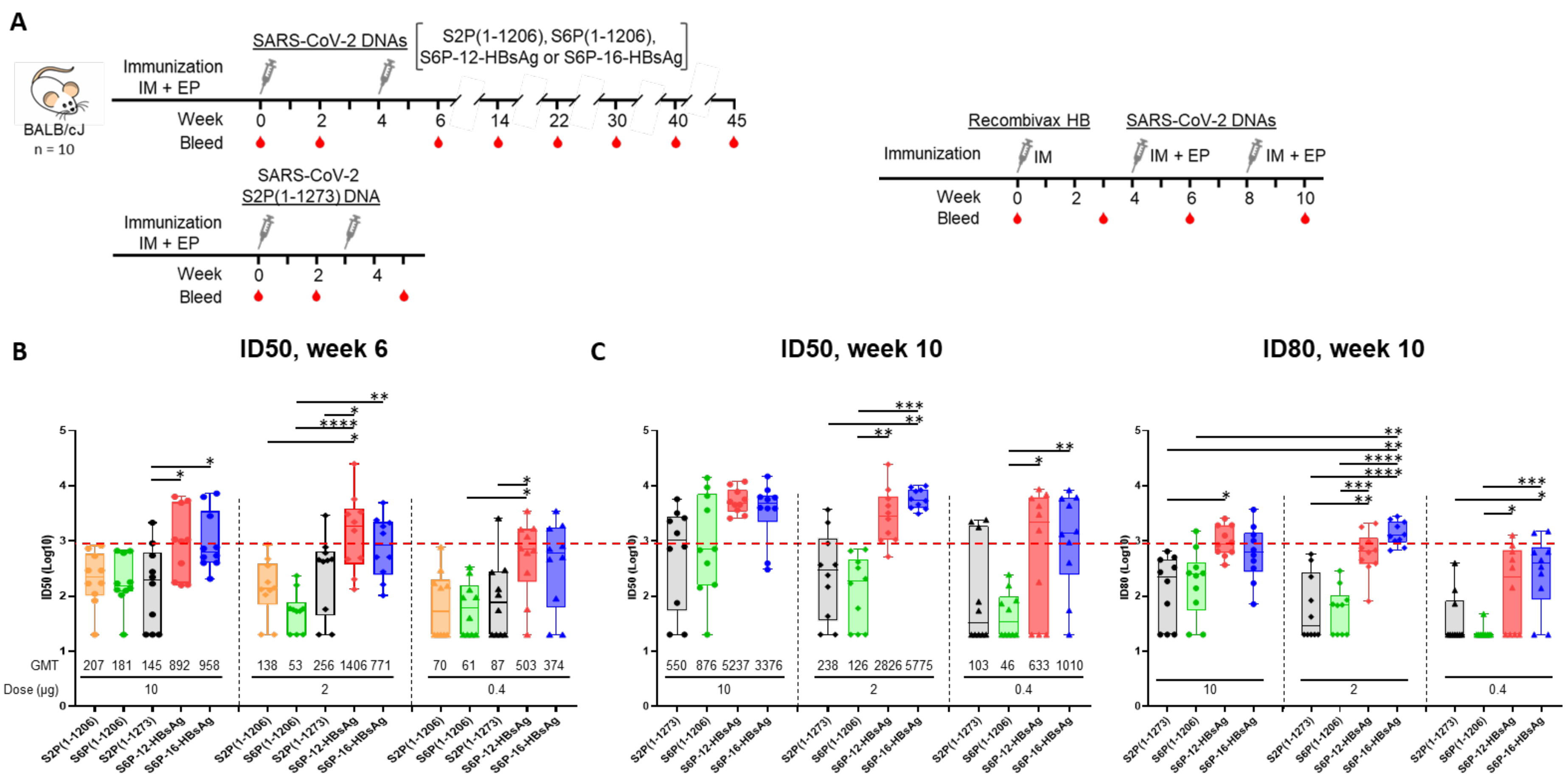

Supplementary Figure 15. Comparison of neutralization potency in mice with and without Recombivax HB pre-vaccination. (A) and (B) were adopted from Fig. 2; (C) was from Fig. 5 and Fig. S12A. The data were plotted in a logarithmic scale in the box and whiskers format. The data points in the median quartile in each group were boxed. Error bars represent 95% confidence interval. The doses and immunogens were indicated below the plots. The data points from each animal were shown as dots, diamonds or triangles for 10, 2, and 0.4 μg doses, respectively. The data points were shown in beige, green, black, red and blue for S2P(1-1206), S6P(1-1206), S2P(1-1273), S6P-12-HBsAg and S6P-12-HBsAg, respectively. The statistical analyses were performed using the two-way ANOVA test. * $p < 0.05$; ** $p < 0.01$; *** $p < 0.001$; **** $p < 0.0001$.
