# Supplementary material for: Self-assembling SARS-CoV-2 spike-HBsAg nanoparticles elicit potent and durable neutralizing antibody responses via genetic delivery
Source: NPJ Vaccines. 2023 Aug 8;8:111. doi: 10.1038/s41541-023-00707-w (PMC10409857; doi:10.1038/s41541-023-00707-w)
Supplement: Supplementary file 2 — REPORTING SUMMARY [file 41541_2023_707_MOESM2_ESM.pdf]

nature portfolio

Corresponding author(s): Richard A. Koup, Amarendra Pegu and John R. Mascola

Last updated by author(s): May 1, 2023

Reporting Summary

Nature Portfolio wishes to improve the reproducibility of the work that we publish. This form provides structure for consistency and transparency in reporting. For further information on Nature Portfolio policies, see our Editorial Policies and the Editorial Policy Checklist.

Statistics

For all statistical analyses, confirm that the following items are present in the figure legend, table legend, main text, or Methods section.

n/a	Confirmed	
☐	☒	The exact sample size (*n*) for each experimental group/condition, given as a discrete number and unit of measurement
☐	☒	A statement on whether measurements were taken from distinct samples or whether the same sample was measured repeatedly
☐	☒	The statistical test(s) used AND whether they are one- or two-sided<br>*Only common tests should be described solely by name; describe more complex techniques in the Methods section.*
☒	☐	A description of all covariates tested
☐	☒	A description of any assumptions or corrections, such as tests of normality and adjustment for multiple comparisons
☒	☐	A full description of the statistical parameters including central tendency (e.g. means) or other basic estimates (e.g. regression coefficient) AND variation (e.g. standard deviation) or associated estimates of uncertainty (e.g. confidence intervals)
☒	☐	For null hypothesis testing, the test statistic (e.g. *F*, *t*, *r*) with confidence intervals, effect sizes, degrees of freedom and *P* value noted<br>*Give P values as exact values whenever suitable.*
☒	☐	For Bayesian analysis, information on the choice of priors and Markov chain Monte Carlo settings
☒	☐	For hierarchical and complex designs, identification of the appropriate level for tests and full reporting of outcomes
☒	☐	Estimates of effect sizes (e.g. Cohen's *d*, Pearson's *r*), indicating how they were calculated

Our web collection on statistics for biologists contains articles on many of the points above.

Software and code

Policy information about availability of computer code

Data collection: Not applicable

Data analysis: Not applicable

For manuscripts utilizing custom algorithms or software that are central to the research but not yet described in published literature, software must be made available to editors and reviewers. We strongly encourage code deposition in a community repository (e.g. GitHub). See the Nature Portfolio guidelines for submitting code & software for further information.

Data

Policy information about availability of data

All manuscripts must include a data availability statement. This statement should provide the following information, where applicable:

- Accession codes, unique identifiers, or web links for publicly available datasets
- A description of any restrictions on data availability
- For clinical datasets or third party data, please ensure that the statement adheres to our policy

All data will be available upon request addressed to Dr. Amarendra Pegu.

Research involving human participants, their data, or biological material

Policy information about studies with human participants or human data. See also policy information about sex, gender (identity/presentation), and sexual orientation and race, ethnicity and racism.

Reporting on sex and gender	Not applicable
Reporting on race, ethnicity, or other socially relevant groupings	Not applicable
Population characteristics	Not applicable
Recruitment	Not applicable
Ethics oversight	Not applicable

Note that full information on the approval of the study protocol must also be provided in the manuscript.

Field-specific reporting

Please select the one below that is the best fit for your research. If you are not sure, read the appropriate sections before making your selection.

☒ Life sciences ☐ Behavioural & social sciences ☐ Ecological, evolutionary & environmental sciences

For a reference copy of the document with all sections, see nature.com/documents/nr-reporting-summary-flat.pdf

Life sciences study design

All studies must disclose on these points even when the disclosure is negative.

Sample size	Sample size was determined based on a power calculation using a free software package (powerandsamplesize.com). In this calculation, the 2 proportions, 2 samples, and 2-sided equation was chosen. Power (1-β) was set at 0.80, and Type I error (α) set at 5%, or 0.05. For nanoparticle groups, the proportions were set at 0.8, i.e. at least 8 out of 10 mice will develop high neutralization titer (> 300 fold of serum dilution) against SARS-CoV-2 pseudoviruses, and the control group proportions were set at 0.25 based on previous data from the VRC on SARS-CoV-2 S2P immunization in mice, i.e. averagely 2.5 mice out of 10 mice will develop high neutralization titer against SARS-CoV-2 pseudoviruses. Sampling ratio was set at 1, with random placement into each group. The sample size is calculated to be 9. Considering potential loss of mice over a long period of time monitoring the durability of antibody responses post vaccination, we used 10 mice each group.
Data exclusions	We did not exclude any data point.
Replication	The ELISA assays in this manuscript were done in duplicates, while the neutralization assays were done in triplicates for each sample.
Randomization	The mice in this study were grouped randomly.
Blinding	The assays were done in blind.

Reporting for specific materials, systems and methods

We require information from authors about some types of materials, experimental systems and methods used in many studies. Here, indicate whether each material, system or method listed is relevant to your study. If you are not sure if a list item applies to your research, read the appropriate section before selecting a response.

Materials & experimental systems

n/a	Involved in the study	
☐	☒	Antibodies
☐	☒	Eukaryotic cell lines
☒	☐	Palaeontology and archaeology
☐	☒	Animals and other organisms
☒	☐	Clinical data
☒	☐	Dual use research of concern
☒	☐	Plants

Methods

n/a	Involved in the study	
☒	☐	ChIP-seq
☒	☐	Flow cytometry
☒	☐	MRI-based neuroimaging

Antibodies

Antibodies used	We used SARS-CoV-2 mAbs that are specific to diverse domains of SARS-CoV-2 spike. A complete list of these mAbs was described in Table S1.
Validation	These mAbs were published and validated by diverse binding and neutralization assays in different labs in the field including the Vaccine Research Center, NIAID, NIH.

Eukaryotic cell lines

Policy information about cell lines and Sex and Gender in Research

Cell line source(s)	Expi293 cells were purchased from Thermo Fisher Scientific.
Authentication	The vials containing the Expi293 cells we have received were labeled correctly. The starting and following cell culture from the vials have behaved normally. We did not authenticate the cell line.
Mycoplasma contamination	Our lab monitors mycoplasma contamination of Expi293 cultures periodically and we found no contamination.
Commonly misidentified lines (See ICLAC register)	Not applicable.

Animals and other research organisms

Policy information about studies involving animals; ARRIVE guidelines recommended for reporting animal research, and Sex and Gender in Research

Laboratory animals	6- to 8-week old BALB/cJ mice from the Jackson Laboratory
Wild animals	*Provide details on animals observed in or captured in the field; report species and age where possible. Describe how animals were caught and transported and what happened to captive animals after the study (if killed, explain why and describe method; if released, say where and when) OR state that the study did not involve wild animals.*
Reporting on sex	Female
Field-collected samples	*For laboratory work with field-collected samples, describe all relevant parameters such as housing, maintenance, temperature, photoperiod and end-of-experiment protocol OR state that the study did not involve samples collected from the field.*
Ethics oversight	The animal studies were performed in compliance with all pertinent US NIH regulations and approval from the Animal Care and Use Committee (ACUC) of the Vaccine Research Center (VRC).

Note that full information on the approval of the study protocol must also be provided in the manuscript.
